# A New N-Substituted 1*H*-Isoindole-1,3(2*H*)-Dione Derivative—Synthesis, Structure and Affinity for Cyclooxygenase Based on In Vitro Studies and Molecular Docking

**DOI:** 10.3390/ijms22147678

**Published:** 2021-07-18

**Authors:** Dominika Szkatuła, Edward Krzyżak, Paulina Stanowska, Magdalena Duda, Benita Wiatrak

**Affiliations:** 1 Department of Medicinal Chemistry, Wroclaw Medical University, Borowska 211, 50-556 Wroclaw, Poland; 2 Department of Inorganic Chemistry, Wroclaw Medical University, ul. Borowska 211a, 50-556 Wrocław, Poland; 3 Student Scientific Club of Medicinal Chemistry, Wroclaw Medical University, Borowska 211, 50-556 Wroclaw, Poland; paulinastanowska4@gmail.com (P.S.); magda.duda12@wp.pl (M.D.); 4 Department of Pharmacology, Faculty of Medicine, Wroclaw Medical University, Mikulicza-Radeckiego 2, 50-345 Wroclaw, Poland; benita.wiatrak@umed.wroc.pl

**Keywords:** phthalimide, arylpiperazine, cyclooxygenase inhibition, molecular docking

## Abstract

Isoindoline-1,3-dione derivatives constitute an important group of medicinal substances. In this study, nine new 1*H*-isoindole-1,3(2*H*)-dione derivatives and five potential pharmacophores were obtained in good yield (47.24–92.91%). The structure of the new imides was confirmed by the methods of elemental and spectral analysis: FT–IR, H NMR, and MS. Based on the obtained results of ESI–MS the probable path of the molecules decay and the hypothetical structure of the resulting pseudo-molecular ions have been proposed. The physicochemical properties of the new phthalimides were determined on the basis of Lipiński’s rule. The biological properties were determined in terms of their cyclooxygenase (COX) inhibitory activity. Three compounds showed greater inhibition of COX-2, three compounds inhibited COX-1 more strongly than the reference compound meloxicam. From the obtained results, the affinity ratio COX-2/COX-1 was calculated. Two compounds had a value greater than that of meloxicam. All tested compounds showed oxidative or nitrosan stress (ROS and RNS) scavenging activity. The degree of chromatin relaxation outside the cell nucleus was lower than the control after incubation with all test compounds. The newly synthesized phthalimide derivatives showed no cytotoxic activity in the concentration range studied (10–90 µM). A molecular docking study was used to determined interactions inside the active site of cyclooxygenases.

## 1. Introduction

The search for new, safe, and effective synthetic drugs is a constant need. Heterocyclic compounds play an important role as the basis of many bioactive molecules. The isoinoline-1,3-dione moiety is of interest to many teams seeking biologically active compounds as candidates for new drugs. 

It has been proven that the phthalimide ring does not cause the side effects characteristic of glutarimide derivatives, and phthalimides are the basis of many products with proven biological activity, including analgesics, anti-inflammatories, and cholinesterases inhibitors used in Alzheimer’s disease [1,2,3,4,5]. Phthalimide analogs exhibit interesting biological activities, including anticonvulsant, antibacterial, antifungal, anti-inflammatory, and analgesic activities [5,6,7,8,9]. 

The mentioned directions of action of isoindoline-1,3-dione derivatives are the result of several mechanisms for various biological purposes. For example, benzylamino-2-hydroxyalkylphthalimide derivatives are described as moderate and balanced multipotent anti-Alzheimer’s agents capable of inhibiting acetcholinesterase, symptomatic target, and binding modifying target (aggregation of β-secretase and β-amyloid) [3,4,5].

While browsing the literature, one can find information about the influence of isoinoline-1,3-dione derivatives on many groups of pro-inflammatory factors, including induced nitric oxide synthase (iNOS), nitric oxide (NO), cyclooxygenase 2 (COX-2), tumor necrosis factor (TNF-), interleukin-1 (IL-1), and interleukin-6 (IL-6). At the same time, they enhanced the expression of anti-inflammatory factors such as arginase-1 and interleukin-10 (IL-10), and inhibited p65 subunit phosphorylation induced by LPS kappa B (NF-B) in macrophages [2].

Some phthalimide derivatives (2-phthalimidoethanol and 2-phthalimide diethyl nitrate) inhibit mechanical allodynia, neurophil recruitment, cytokine and chemokine production in murine model of articular inflammation [10], and exhibit activity in experimental models of inflammatory and neuropathic pain (N-3-hydroxypropylphthalimide and N-carboxymethyl-3-nitrophthalimide) [11]. 

The anti-inflammatory activity of N-alkyl-3,5-dihydroxyphthalimide and N-alkyl/aryloalkyl-3,5-dimethoxyphthalimide was also found associated with the suppression of the Toll-like receptor (TLR4) signaling pathway by down–regulating the activation of interferon regulatory factor 3 (IRF-3) and interferon-β and signal transducer expression [9]. Some exo-N-hydroxy-7-oxabicyclo [2.2.1] hept-5-ene-2,3-dicarboximide derivatives, containing the pharmacophore 2-hydroxypropyl-4-arylpiperazine were tested as compounds with high specificity and affinity toward serotoninergic receptors. 

It was found that the structural elements: the heterocyclic moiety, the hydroxyalkyl linker and the 4-substituted piperazine determine the high activity and affinity for 5-HT_1AR_ receptors [12]. The significant reactivity of the acid proton at the imide nitrogen atom allows for the preparation of many phthalimide derivatives, and the type of pharmacophore introduced into the molecule determines the direction of action of the new derivatives [13,14,15,16,17].

N-alkyl-isoindoline-1,3-diones derivatives showed good inhibitors of the COX enzyme, as did indomethacin and celecoxib [18]. The presence of the aromatic moiety is important for the affinity for COX2. The introduction of a nitrogen atom (piperazine ring) into the N-methylphthalimide ligand increases the lipophilicity and affinity for many amino acids of the COX enzyme in both isoforms. In COX-2 molecular docking studies, the new imides showed strong interactions with many amino acids, heralding potential blocking effects for these compounds, but further biological activity studies are needed [18].

Research on 3,4-pyridinedicarboximide derivatives allowed to establish the relationship between the analgesic effect of the new pyrrolo[3,4-c]pyridine-1,3(2*H*)-dione derivatives and the structure of the alkyl linker and the amine part [19,20]. It has been shown that the presence of the 2-hydroxypropyl-4-arylpiperazine pharmacophore attached to the nitrogen atom of the cyclic imide may be responsible for the analgesic properties stronger than that of aspirin, comparable to morphine [13,19,20].

Other reports show that phthalimide derivatives in which the pharmacophore is preceded by an oxygen atom have shown another activity [12]. This confirms that even slight modifications to the structure of the molecule can significantly change the direction of action and biological purpose of the designed synthetic drugs.

Based on the above reports, the synthesis of new phthalimide derivatives being the subject of this study was designed. To establish the role of the imide moiety, the ring of the appropriately substituted pyridine was replaced with a benzene ring. A group of phthalimide derivatives (1,2-benzenedicarboximide) was obtained, which are analogs of the previously described 3,4-pyridinedicarboximide derivatives [19,20]. Moreover, a modification of the structure of the alkyl linker between the imide and the arylpiperazine was introduced. The additional oxygen atom of the carbonyl group was supposed to lower the basicity of the piperazine nitrogen atom, allowing the formation of another hydrogen bond between the product molecule and the biological target [21].

Compounds have been designed with potential analgesic and anti-inflammatory effects by blocking cyclooxygenase (COX) activity. It is an enzyme that catalyzes the conversion of arachidonic acid to prostanoids: prostaglandins, prostacyclin, and thromboxane [22]. Two major isoforms of the COX enzyme have been identified: COX-1 (constitutional) and COX-2 (inducible). It was assumed that inhibition of COX-2 was responsible for anti-inflammatory and analgesic effects. Previously, blocking COX-1 was thought to cause adverse effects and disrupt homeostasis. It is now known that even selective COX-2 inhibitors can cause serious cardiovascular toxicity [23]. Therefore, the search for new cyclooxygenase inhibitors that will allow for safer pharmacotherapy of pain-related inflammation is still valid. 

In our research, molecular docking of the obtained phthalimide derivatives to cyclooxygenase and protein isoforms was performed, which was compared with the results of affinity for enzymes using the in vitro method. The next step will be to compare the activities of 3,4-pyridinedicarboximide analogs and phthalimides in animal studies. This will allow us to understand the role of the imide moiety in the appearance of analgesic properties of new derivatives.

## 2. Results and Discussion

### 2.1. Chemistry

In order to compare the biological properties of 3,4-pyridinedicarboximide and phthalimide as basic systems, it was decided to synthesize new isoindoline-1,3-dione derivatives as analogs previously described compounds with confirmed analgesic activity, containing the following amino residues: 1-phenylpiperazine (**A**), 1-(2-methoxyphenyl)piperazine (**B**), 1-(3-trifluoromethphenyl)piperazine (**C**), and as a supplement 4-benzhydrylpiperazine/4-diphenylmethyl-1-piperazine (**D**) [24,25]. 

**Scheme 1:**
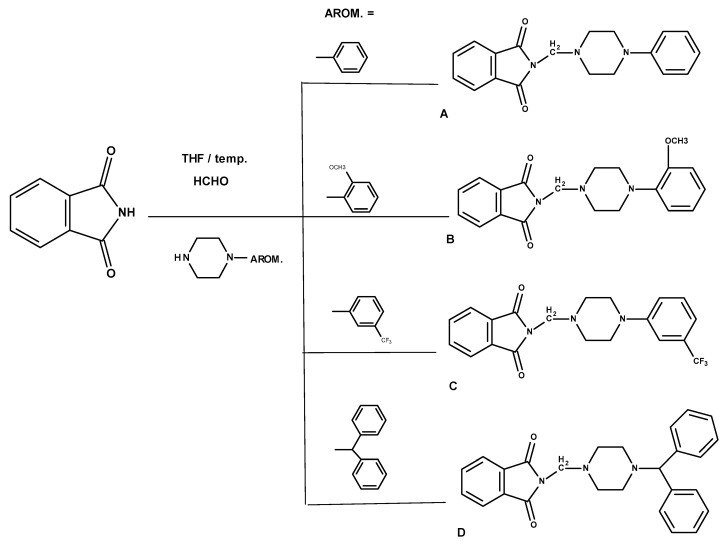
Synthetic scheme of the studied compounds **A**–**D**.

The presence of an acidic proton at the 2-position of the imide nitrogen allows for the execution of the aminomethylation reaction according to the mechanism described by Mannich [26]. The reaction was carried out using an aqueous solution of formaldehyde (HCHO) and N-arylpiperazines (commercial Sigma-Aldrich) at the boiling point of tetrahydrofuran (THF) for several hours (Scheme 1). TLC monitored the course of the condensation. After cooling the reaction mixture, crystallized final compounds **A**–**D** were obtained, which were filtered on a Bűchner funnel. After drying, the products were recrystallized from ethanol (**B**, **C**) or a mixture of ethanol and hexane (**A**) or cyclohexane (**D**). ^1^H NMR spectral analysis was performed in chloroform (Deuterochloroform: CDCl_3_ for NMR). All reactions proceeded with a good yield of 47.26–92.91%. 

**Scheme 2:**
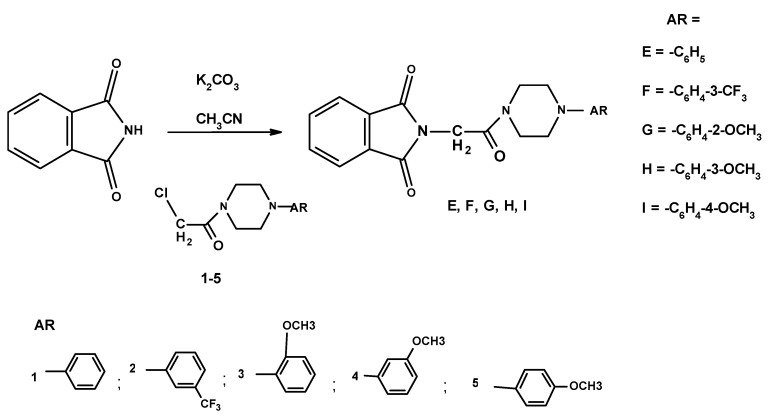
Synthesis of N-acethylaminoaryl derivatives **E**–**I**.

The second group of new imides was planned as N-acetylpiperazine aryl derivatives of isoindoline-1,3-dione. The structure of the new **E**–**I** phthalimides meets the assumptions of the optimal structure of the pharmacophore group for the expected analgesic activity proposed by Sahin et al. [21]. The cyclic imide moiety is linked to the arylpiperazine residue by a linker—(CH_2_)_n_–(C=O)-. The structure of the new compounds is shown in Scheme 2. 1-Chloroacetyl-4-aryl-piperazine derivatives **1**–**5** were obtained according to the procedure proposed previously [21,27,28].

**Scheme 3:**
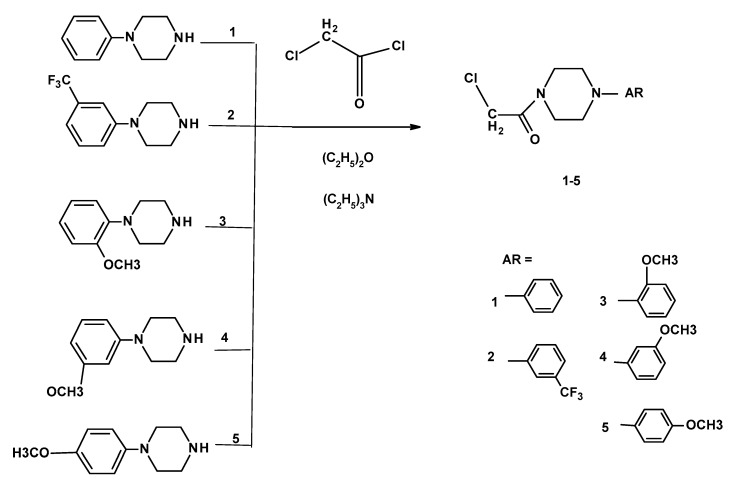
Method of synthesis of 1-chloroacetyl-4-aryl-piperazine derivatives **1**–**5**.

To obtain the **E**–**I** derivatives, the commercial form of phthalimide (Sigma-Aldrich) was used, which was condensed with N-arylpiperazine acyl chlorides (**1**–**5**) [21,27,28] prepared as previously described. First, acetamide derivatives (**1**–**5**) were prepared: 1-chloroacetyl-4-phenylpiperazine (**1**), 1-chloroacetyl-4-(3-trifluorophenyl) piperazine (**2**) [27], -chloroacetyl-4-(2-/3-(4-methoxyphenyl) piperazine (**3, 4,** and **5,** respectively). The synthesis involved N-acylation of N-arylpiperazines by dropwise addition of chloroacetyl chloride in diethyl ether solution to a mixture of the appropriate N-arylpiperazine, diethyl ether and triethylamine to obtain derivatives **1**–**5** without any by-product. The synthesis of intermediates **1**–**5** is outlined in Scheme 3. Commercial phthalimide (Sigma-Aldrich) was then alkylated with intermediates **1**–**5** in acetonitrile in the presence of potassium carbonate, which provided the target compounds (**E**–**I**).

**Scheme 4:**
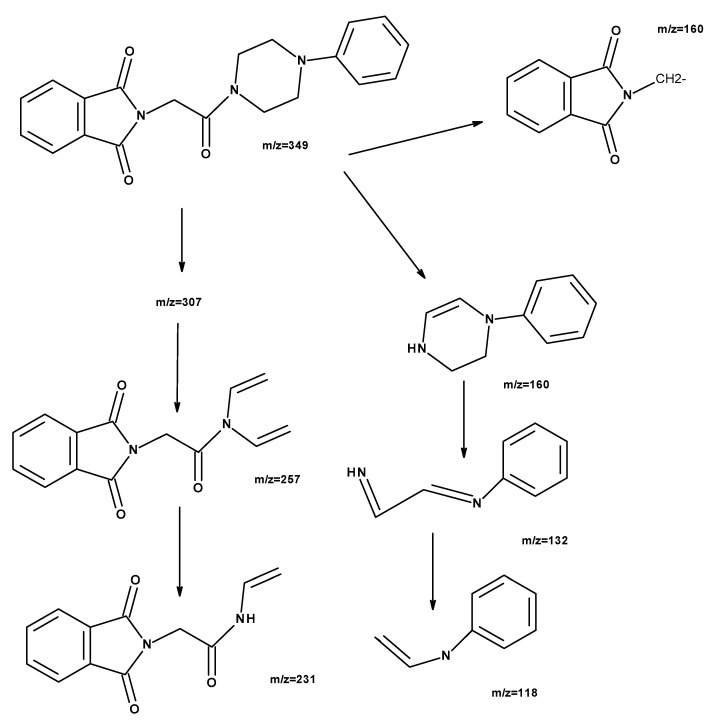
The probable mechanism of E degradation in methanol solution proposed based on ESI–MS.

**Scheme 5:**
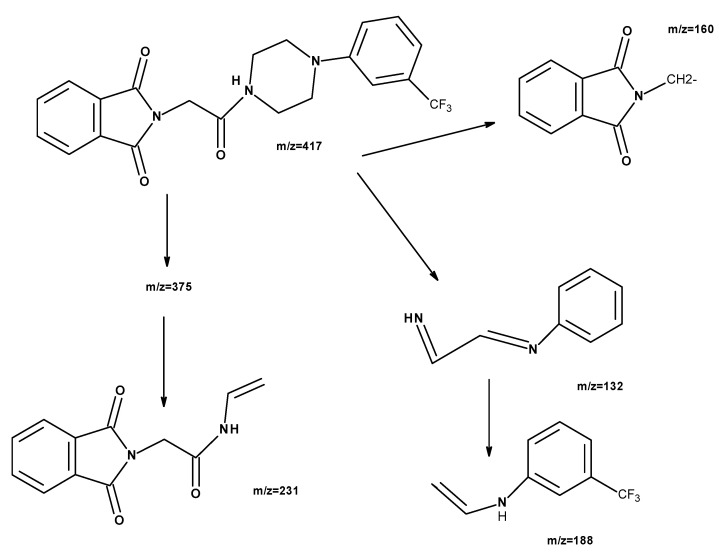
The probable mechanism of F degradation in methanol solution proposed based on ESI–MS.

**Scheme 6:**
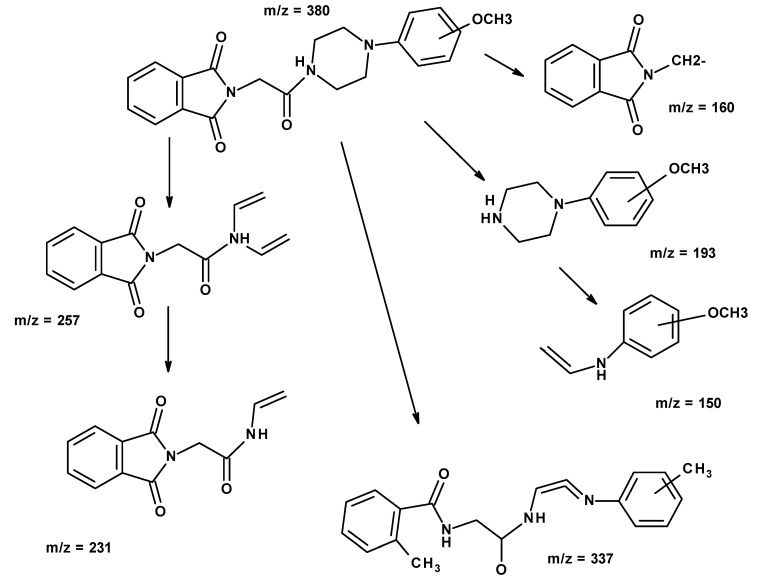
The probable mechanism of G, H, and I degradation in methanol solution, proposed based on ESI–MS.

The final compounds had a sharp melting point (m.p.), correct elemental analyses (C, H, N), IR and ^1^H NMR spectra and mass values determined by MS–MS. In some cases (**E**–**I**), the daughter ions formed during the fragmentation of the final compound have been identified by electrospray ionization–mass spectrometry (ESI–MS). The structures of degradation products and the mechanics of their formation were designed in accordance with the information on the mass of quasi-molecular ions and fragmented degradation products using the DataAnalysis Program [29]. The structure of the degradation products and the mechanisms of their formation were deduced based on information on the mass of quasi-molecular ions and fragmented degradation products based on previous information and literature reports [27,30]. The chromatogram of the sample solution and the proposed **E**–**I** imide degradation pathways are shown in Scheme 4, Scheme 5 and Scheme 6 and in additional materials: I (**E**), II (**F**), III (**G**), IV (**H**), and V (**I**). The characteristics of **E**–**I** degradation products are included in Tables S1–S5 in Supplementary Materials.

The structure of the compound **E** was confirmed based on the MS spectrum, which showed the presence of the molecular ion [L + H]^+^ with *m/z* = 350.1479 being the main peak, which corresponds to the molecular weight of this compound. The spectrum also includes additional peaks, derived from adducts of the molecule with the sodium ion [L + Na]^+^ *m/z* = 372.1300 and the dimer of the molecule with the sodium ion [2L + Na]^+^ *m/z* = 721.2724. With the help of the DataAnalysis Program [29], a computer simulation was performed for the expected compound and its adducts, and the value error between *m/z* found and *m/z* calculated was determined. The MS spectrum analysis data above for the compound is summarized in the table (I, Table S1).

The structure of compound **F** was confirmed based on the MS spectrum, which showed the presence of the molecular ion [L + H]^+^ with *m/z* = 418.1357 being the main peak, which corresponds to the molecular weight of this compound. The spectrum also includes additional peaks, derived from adducts of the molecule with the sodium ion [L + Na]^+^ *m/z* = 440.1176 and the dimer of the molecule with the sodium ion [2L + Na]^+^ *m/z* = 857.2485. A computer simulation was performed for the expected compound and its adducts, and the value error between *m/z* found and *m/z* calculated was determined (II, Table S2). The proposed course of the degradation of the **F** molecule is shown in Scheme 5. 

The structure of the methoxyphenyl derivatives **G**–**I** molecules was confirmed based on the MS spectrum, which showed the presence of the molecular ion [L + H]^+^ *m / z* = 380.1575 (**G**), 380.1573 (**H**), and 380.1630 (**I**), a peak that corresponds to the molecular weight of these compounds. There is also an additional peak in the spectrum, coming from the adducts of the molecule with the sodium ion [L + Na]^+^ *m/z* = 402.1391 (H), 402.1419 (I) and the dimer of the molecule with the sodium ion [2L + Na]^+^ *m/z* = 781.2925 (G), 781.2912 (H), and 781.2959 (I). The proposed course of the degradation of the methoxy derivative molecule is shown in Scheme 6.

Using the DataAnalysis Program [29], a computer simulation of the expected compound and its adducts was performed, and the error in the value between the *m/z* found and the calculated *m/z* was determined. The above MS spectral analysis data for the compound is presented in the supporting materials in Tables S3–S5.

Possible daughter ion structures have been suggested earlier. The instability of the piperazine ring has been previously described for fluoroquinolone derivatives. Not all fragmentation products have been solved (Scheme 4, *m/z* = 307; Scheme 5, *m/z* = 375), for some, they have not been confirmed in the literature [27,30,31,32,33,34,35,36,37].

### 2.2. Molecular Properties

The bioavailability of compounds **A**–**I** was assessed using Lipiński’s “rule of five” [38]. It is described by analyzing the physical properties of the molecule, such as molar weight (MW), polar surface area (PSA), hydrophobicity (logP), and the number of the donor (H-d) or acceptor (H-a) sites of nature of hydrogen bonds. According to this principle, drugs that are well absorbed after oral administration are those that meet the criteria: molar mass below 500 Da, less than five sites of hydrogen bond donors (H-d), less than 10 hydrogen bond acceptor sites (H-a), logP (log octanol: water partition coefficient) less than 5, PSA below 90 Å^2^ for compounds that cross the blood–brain barrier well [38,39,40].

As the sum of the polar surfaces of all atoms, the polar surface of a molecule indicates the probability of a molecule overcoming biological barriers. The Topological Polar Surface Area (TPSA), according to the methodology described by Ertl et al. [40], is the sum of the areas occupied by oxygen and nitrogen atoms and hydrogen atoms attached to them, i.e., it is a potential for the formation of hydrogen bonds of the molecule. Therefore, the TPSA value can be considered an indicator of drug absorption and the ability to overcome biological barriers—including absorption from the gut and crossing the blood–brain barrier. The values describing the **A**–**I** derivatives are included in Table 1. Log*P* values must be in the range of −0.4 to +5 [39,40]. The obtained values logP of **A**–**I** (2.11, 2.37, 3.13, 3.14, 3.25, 4.0, 4.61) indicate a high probability of absorption after oral administration (calculated adopting the methodology developed by Molispiration [41]. 

All new phthalimide derivatives (**A**–**I**) meet the requirements of Lipiński’s “rule of five.” However, the TPSA value from 45.55–70.16 Å^2^ may indicate good absorption after oral administration (TPSA < 140 Å^2^) and the ability to cross the blood–brain barrier (TPSA < 90 Å^2^) [38,39,40,42].

### 2.3. Biological Evaluation

The newly synthesized phthalimide derivatives showed no cytotoxic activity in the concentration range studied (10–90 µM, Table 2). The methoxy substituent, regardless of the phenyl substitution site (*ortho*-, *meta*-, or *para*-), causes the death of 50% of the NHDF cells at a concentration just above 90 µM. The calculated IC50 values were the lowest for compound **D**, 90.28 µM. 

All tested compounds show a stronger affinity for COX-2 than for COX-1 (Table 2). In addition, compounds **E, F**, **H**, and **I** showed greater inhibition of COX-2 than the reference compound meloxicam. On the other hand, compounds **B**, **D,** and **E** showed greater inhibition of COX-1 than meloxicam. The COX-2/COX-1 ratio informs about the selectivity of the tested compounds towards COX-1 and COX-2—the activity of which cyclooxygenase is more strongly inhibited. Based on the obtained results, the COX-2/COX-1 ratio was calculated, and only compounds **B** and **D** have a value greater than that of meloxicam. Compounds **A**, **C**, **E**, **F**, **G**, **H,** and **I** are COX-2 selective. At the same time, it should be noted that the selectivity of compound **E** is comparable to that of meloxicam. Interestingly, compounds **F** and **H** show nearly three times greater selectivity towards COX-2 than the reference compound—meloxicam. Meloxicam was used as the reference compound in the study as it is COX-2 selective.

As a consequence of metabolic changes, reactive forms of both oxygen and nitrogen (ROS and RNS) are formed, which can cause oxidative or nitrosan stress, respectively. It may be a consequence of disease processes or cell proliferation, e.g., during regeneration of damaged tissues. The level of RONS may also be influenced by cyclooxygenases 1 and 2. At the same time, an increase in the level of RONS and COX may affect DNA damage (Table 3.). Therefore, the influence of the tested compounds on the level of RNS, ROS, and the level of DNA strand damage after the previous 24-h preincubation with 50 µM LPS was assessed. 

All compounds tested showed both ROS and NO scavenging activity. The lowest NO concentrations were observed after incubation with compounds **B**, **G**, **H,** and **I**, where the NO concentration was about 4 pg/mL. The highest scavenging of ROS was observed after incubation with compounds **G**, **H,** and **I**—approximately 29.0% compared to control. The amount of chromatin relaxation outside the cell nucleus was lower after incubation with all test compounds than the control. The observed ROS and NO scavenging activity was highest after incubation of cell cultures preincubated with LPS when COX-2 selective compounds were used. At the same time, the strongest regeneration of DNA strand breaks was observed after these compounds. On this basis, it can be assumed that compounds **G**, **H,** and **I** exhibit anti-inflammatory activity dependent on the inhibition of COX-2. 

### 2.4. COX-1 and COX-2 Molecular Docking Study

**Figure 1:**
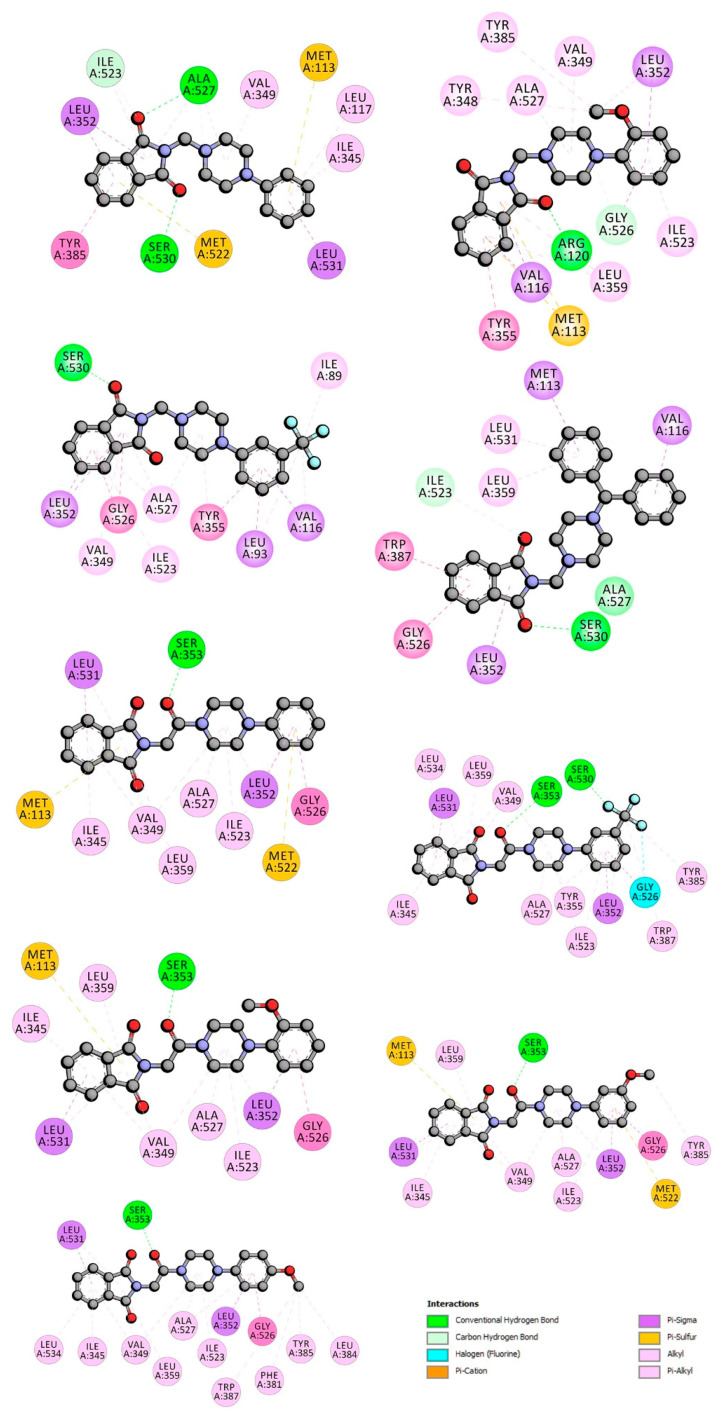
2D interaction plot of studied compounds with COX-1.

**Figure 2:**
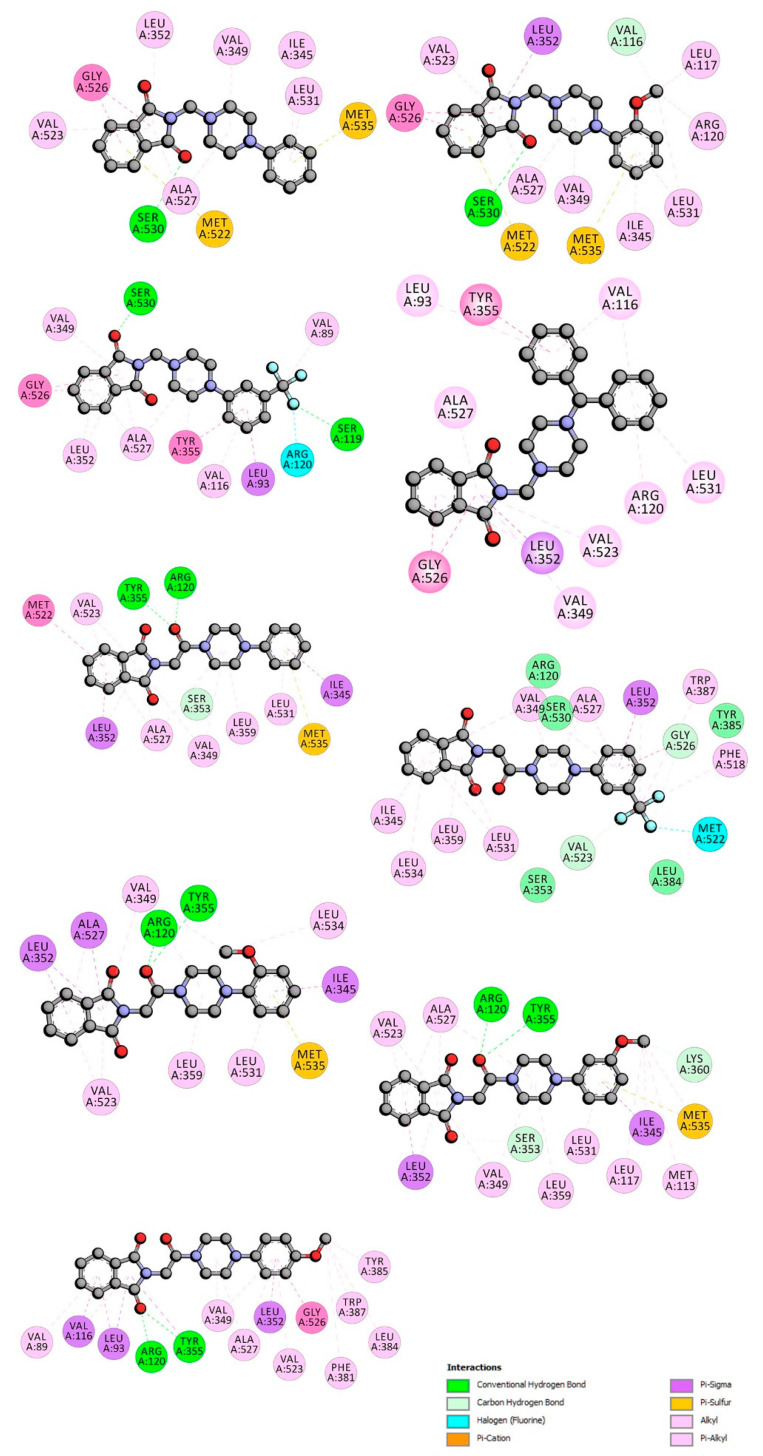
2D interaction plot of studied compounds with COX-2.

**Figure 3:**
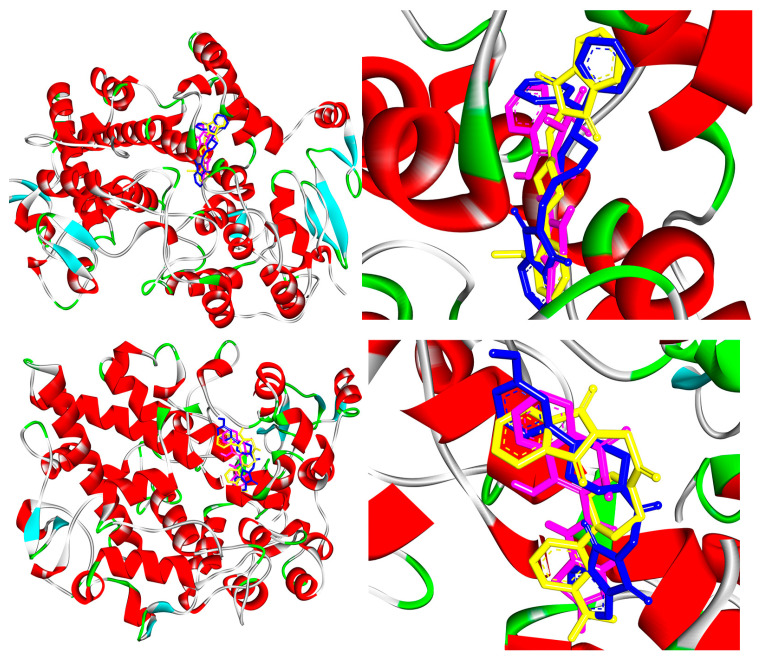
Docking poses of compounds **B** (yellow), **D** (blue), and meloxicam (red) inside COX-1 active site (**above**) and docking poses of **F** (yellow), **H** (blue), and Meloxicam (red) inside COX-2 active site (**bottom**).

To investigate the possible binding interactions of synthesized compounds inside active site molecular docking study was performed. The theoretical binding free energy (ΔG°) and inhibit constant (K_i_) are listed in Table 4. The ΔG° is negative for all compounds for both COX-1 and COX-2 interactions. The interactions with COX-2 are stronger. Studied compounds are relatively large, making it more difficult to dock into a smaller COX-1 pocket. The lowest value was found for **G** and **E**. However, the differences are not spectacular. All compounds bind to COX-1 and COX-2 well. This result is in good correlation with biological evaluation, where all compounds inhibition of COX-1 and COX2. An important role in interactions with both cyclooxygenases plays hydrogen bonds: Ser530, Ser353, Agr120, and Ala527 with O (for COX-1), and Ser530, Arg120, and Tyr355 with O (for COX-2). Also, various types of π interactions with rings are observed. Details are presented in Figure 1 and Figure 2. In Figure 3, the locations of the most active compounds **B**, **D** (against COX-1) and **F**, **H** (against COX-2, with the high COX-2/COX-ratio) compared to meloxicam are presented. The studied compounds are situated in subdomain B [28] with a similar orientation to meloxicam. 

According to the biological evaluation, compound **D** is the most potent COX-1 inhibitor. As shown in Figure 1, a hydrogen bond between oxygen atom from the carbonyl group and Ser530 is formed (distance 2.5 Å). The isoindoline-1,3-dione part of the compound is involved in interaction with Trp387 (π-π), Gly526 (amide-π stacked) and Leu352 (π-σ). The two benzene rings of the amine part interact with Val116, Met113 via π-σ contact and π-alkyl interactions with Leu539 and Leu531 residues. As mentioned above, the location is similar to meloxicam (Figure 3). In general, the COX binding site has four characteristic subdomains I–IV [28]. Subdomain 1 represents the mode of binding of flurbiprofen; subdomain 2 represents the mode of binding of meloxicam and piroxicam; subdomain 3 represents an entrance region of the enzyme binding domain, and subdomain 4 represents the position of the residue in position 523. Compound **D** takes position in region 2 like meloxicam (Figure 3). Compound **D** is active also against COX-2 but compounds **E**–**I** with longer linker between isoindole-1,3(2H)-dione part and arylpiperazine residue, showed greater inhibition of COX-2 (Table 2). The most potent COX-2 inhibitor, compound **H**, interacts with Arg120 and Tyr355 via hydrogen bond with the carbonyl group from the linker (distance 2.6 Å and 2.2 Å). The molecule is well fitted into the COX-2 binding site and it is stabilized by several hydrophobic interactions (Figure 2). The isoindole-1,3(2H)-dione part of the compound is involved in interaction with Leu352 (π-σ), Ala523, and Val523 (π-alkyl). The piperazine ring interacts with Val349, Leu359 (alkyl), and the phenyl group via π-alkyl interactions with Ile345 and Leu531 and methoxy group with Leu117 and Met113 (alkyl). Compound **H** into the binding pocket occupying a similar region as meloxicam in region II (Figure 3). 

The comparison of ΔG°, K, and inhibiting activity for **A**-**C**, **B**-**G**, and **C**–**F** pairs shows the effect of the longer linker between isoindole-1,3(2H)-dione part and arylpiperazine residue. For the **A**-**E** pair, with unsubstituted phenyl, compound **E** with (CH_2_)–(C=O)- linker, shows a more negative value for ΔG°, lower K_i_ (Table 4), and also greater inhibition of COX-1 and COX-2 (Table 2). The same effect is observed for pair **B**–**G**, compounds with methoxy group in aryl ring. However, only for COX-2, the stronger interactions (more negative ΔG°) increase the biological activity. The more negative ΔG° for C and G could be a result of additional interaction with the carbonyl group from the linker (Figure 1 and Figure 2). For **C**-**F**, with trifluoromethyl group in the phenyl, longer linker increases (less negative) the theoretical binding free energy for COX-1, but for COX-2 the interactions with **F** are stronger.

Compounds **E**, **F**, and **H** have different substituent into the *meta* position of the benzene ring: -H, -CF_3_, and -OCH_3_, respectively. The additional group in the aromatic ring seems to be unfavorable. The binding free energy is less negative and the inhibit constant is greater (Table 4). 

Compounds **G**, **H**, and **I** have the same -OCH_3_ group in phenyl but into a different position. The lowest ΔG° was calculated for compound **G**, with methoxy group into position *ortho*. Positions *meta* and *para* reduce the forces of the interaction, especially for COX-1 (Table 4). The inhibition constant increases: *ortho* < *meta* < *para*. The position into the binding site is very similar for **G**, **H**, and **I**, but the methoxy group in position *meta* and *para* can interact with Tyr385 and Leu384 residues (Figure 1 and Figure 2).

A molecular docking study showed that **G** is the compound with the lowest free binding energy. As can be observed, it occupies a close area, similar to that observed for meloxicam, where the following structural correspondences occur: thiazine ring with arylpiperazine fragment, carboxamide with (CH_2_)–(C=O), and thiazole with phthalimide fragment. For COX-2, compound **H** interacts with Arg120 and Tyr355 via hydrogen bond with carbonyl group from (CH_2_)–(C=O) moiety (distance 2.3 Å, 2.5 Å). The isoindol-1,3(2H)-dione part of the compound is involved in interaction with Leu352, Ala523 (π-σ), and Val523 (π-alkyl). The piperazine ring interacts with Val349, Leu359 (alkyl), phenyl group via π-alkyl interactions with Leu531, π-σ with Ile345, π-sulfur with Met535, and methoxy group with Leu534 (Figure 2). These interactions are very similar to compound **H**, the most active in biological evaluation. Compared to meloxicam, compound **G** (or **H**) interacts directly with Arg120 and Tyr355, meloxicam (or other oxicams) do not directly interact by hydrogen bonding. It interacts indirectly by bridging with two tightly bound water molecules: a tetrahedrally coordinated water bound to Arg-120 and Tyr-355 [43]. A hydrogen bond between the 4-hydroxyl group of meloxicam and Ser 530 is reported [43]. It seems to be similar to hydrogen bonding between the carbonyl group and Ser353 observed into the binding pocket of isomer COX-1. The subpocket with Leu531 and Ile345 plays an important role in the interaction of meloxicam with COX-2 [43]. Contacts with thiazine moiety stabilize the complex. As seen in Figure 2, compound **G** interacts with Leu531 and Ile345 by benzene ring. The worst derivative, with the highest energy, is compound **C.** The linker without a carbonyl group removes interaction with Arg120 and Tyr355 (hydrogen binding). CF_3_ group in phenyl (instead OCH_3_) removes interaction with Leu531 and Ile345.

## 3. Materials and Methods

### 3.1. Chemistry

All the results of C, H, and N determinations (carried out by Carlo Erba (Waltham, MA, USA) Elemental Analyzer Model NA 1500) were within ±0.4% of theoretical values. All the melting points were uncorrected. FTIR spectra were ran on Perkin-Elmer Spectrum Two, UATR FT–IR spectrometer (Perkin Elmer, Waltham, MA, USA). The samples were applied as solid.

^1^H NMR spectra were determined in CDCl_3_ on Bruker (Billerica, MA, USA) Ultra Shield 300 MHz using tetramethylsilane as an internal standard (IS).

Mass spectrometry (ESI–MS). ESI–MS experiment was performed on a compact^TM^ mass spectrometer (Bruker Daltonics, Bremen, Germany) equipped with a standard ESI source. The instruments were operated in the positive-ion mode. Spectra were recorded for samples dissolved in MeOH.

Phthalimide 99% was from Acros Organic (Fair Lawn, NJ, USA). 1-Phenyl-4-piperazine (>98%, C_10_H_14_N_2_, MW = 162.24, MP = 17–19 °C; Alfa Aesar, Ward Hill, MA, USA); 1-(2 or 3/4-methoxyoheny)piperazine (>98%, C_11_H_16_N_2_O, MW = 192.26); 1-(3-trifluoromethyl)phenylpiperazine, (98%, C_11_H_13_F_3_N_2_, MW = 230.21; MP = 65–71 °C; Alfa Aesar, Ward Hill, MA, USA), 1-Diphenylmethyl-piperazine (98%,C_17_H_20_N_2,_ MW = 252.36, MP = 90–93 °C; Fluka Ward Hill, MA, USA).

#### 3.1.1. General Method for the Preparation of the Mannich Bases **A**–**D**

A 1.0 g (0.0068 mol) of 1*H*-isoindolo-1,3(2*H*)-dione was dissolved in 40 mL of tetrahydrofurane, and to this suspension, 1 mL of 33% formaline was added. This mixture was refluxed for 0.5 h. After this time, 0.007 mol of suitable N-arylpiperazines were added, again refluxed for 10 h. After one hour, this mixture was cleared, and after 2 more hours, the products started to precipitate. TLC monitored the reactions. The separated solid substance was collected on a filter and washed with diethyl ether, and dried (crude). The analytical samples were obtained after crystallization from ethanol (**A, B, C**) or ethanol and n-hexane(1:1) (**D**).

The properties of obtained compounds **A**–**D** show below in Table 5.

^1^H NMR plots are available in supplementary file.

**A**: N-[(4-phenyl-1-piperazinyl)methyl]-1*H*-isoindole-1,3(2*H*)-dione:

^1^H NMR w CDCl_3_ δ [ppm]: 2.71–3.0 (distorted t-4H, -CH_2_-N-(C**H_2_**)**_2_**-); 3.07–3.35 (distorted t-4H, -(C**H_2_**)**_2_**-N-C_6_H_5_); 4.73 (s- 2H, =N-C**H_2_**-N=); 6.89–7.25 (m- 5H, **H** aromatic of phenyl); 7.74–7.77 (m- 2H, **H** aromatic at C3 and C6 of phthalimide); 7.87–7.9 (m- 2H, **H** aromatic at C4 and C5 of phthalimide).

**B**: N-[(4-(2-methoxyphenyl)-1-piperazinyl)methyl]-1*H*-isoindole-1,3(2*H*)-dione:

^1^H NMR w CDCl_3_ δ [ppm] of A: 2.82–3.35 (distorted m-8H, -CH_2_-N-(C**H_2_**)**_2_**-) + -(C**H_2_**)**_2_**-N-C_6_H_4_ –OCH_3_); 3.81 (s- 3H, -OCH3), 4.76 (s- 2H, =N-C**H_2_**-N=); 6.81–6.92 (m- 4H, **H** aromatic of phenyl); 7.75–7.77 (m- 2H, **H** aromatic at C3 and C6 of phthalimide); 7.87–7.9 (m- 2H, **H** aromatic at C4 and C5 of phthalimide).

**C:** N-[(4-(3-trifluoromethyl)phenyl)-1-piperazinyl)methyl]-1*H*-isoindole-1,3(2*H*)-dione:

^1^H NMR w CDCl_3_ δ [ppm]: 2.75 -3.0 (distorted t-4H, -CH_2_-N-(C**H_2_**)**_2_**-); 3.19–3.45 (t-4H, -(C**H**_2_)_2_-N-C_6_H_4_ –mCF_3_); 4.74 (s- 2H, =N-C**H_2_**-N=); 7.0–7.07 (m- 3H, H aromatic of phenyl at C2, C4, C6); 7.29–7.35 (m-1H, H aromatic of phenyl at C-5); 7.74–7.76 (m- 2H, H aromatic at C3 and C6 of phthalimide); 7.87–7.9 (m- 2H, H aromatic at C4 and C5 of phthalimide).

**D**: N-[(4-(diphenyl-methyl)-1-piperazinyl)methyl]-1*H*-isoindole-1,3(2*H*)-dione:

^1^H NMR w CDCl_3_ δ [ppm]: 2.30–2.55 (distorted t-4H, -CH_2_-N-(C**H_2_**)**_2_**-); 2.65–2.87 (distorted t-4H, -(C**H_2_**)**_2_**-N-CH-(C_6_H_5_)_2_); 4.20 (s-1H, =N-C**H**-(C_6_H_5_)_2_); 4.67 (s- 2H, =N-C**H_2_**-N=); 7.15–7.37 (m- 10H, **H** aromatic of phenyl); 7.79–7.80 (m- 2H, **H** aromatic at C3 and C6 of phthalimide); 7.89–7.90 (m- 2H, **H** aromatic at C4 and C5 of phthalimide).

#### 3.1.2. General Method for the Preparation of the 2-chloro-1-(N-arylopiperazinyl)ethanone **1**–**5**

1-Phenyl-4-piperazine (**1**) (1.62 g, 0.01 mol)/ 1-(3-trifluoromethyl)piperazine (**2**) (2.3 g; 0.01 mol)/1-(2,3 or 4–methoxyphenyl)piperazine (**3**, **4**, **5**)(1.62g, 0,01 mol) and triethylamine (1.4 g; 0.013 mol) were dissolved in 100 mL of diethyl ether. Chloroacetyl chloride (1.13 g, 0.01 mol) in 35 mL of diethyl ether was added slowly over the course of 1 h. The mixture was allowed to stir at room temperature for 30 min; after this time the solvent was evaporated completely under reduced pressure, and the residue was dissolved in 120 mL of CHCl_3_ and 20 mL of water. The organic layer was separated, dried (Na_2_SO_4_), filtered gravitationally using a corrugated filter paper and concentrated. The residue was crystallized from ethanol (**1**,**3**,**4**,**5**). The oil residue (**2**) was purified by a chromatography column (silica gel mesh 70–230, ethyl acetate, Rf = 0.79, monitoring by TLC). The structure of intermediates **1–5** obtained was confirmed by elemental analysis (C, H, N) and ^1^H NMR spectral, MS-MS.

2-Chloro-1-(4-phenyl-1-piperazinyl)ethan-1-on (**1**): 

C_12_H_15_N_2_OCl (MW 238.71; MP = 76–78 °C); yield 68.76%; 1H-NMR (300 MHz, CDCl3), δ (ppm) = 3.19–3.32 (t-4H, -CO-N-(CH2)2-); 3.71–3.88 (t-4H, -(CH2)2-N-C6H4-), 4.13 (s-2H, -CH2-CO-); 6.94–7.07 (m-3H, H aromatic at C3, C4, C5 of phenyl); 7.27–7.46 (m-2H, H aromatic at C2, C6 of phenyl); MS-MS[ppm] = [L + Na^+^] 261.0765, found 261.0753 (4.60); [2L + Na^+^] 499.1638, found 499.1639 (0.2);

2-Chloro-1-[4-(3-fluoromethyl)phenyl-1-piperazinyl]ethan-1-on (**2**): 

C_13_H_14_N_2_OF_3_Cl (MW 306.71); yield 80.72%; 1H-NMR (300 MHz, CDCl3), δ (ppm) = 3.24–3.38 (t-4H, -CO-N-(CH2)2); 3.71–3.88 (t-4H, -(CH2)2-N-C6H4-); 4.14 (s-2H, -CH2-CO-), 7.10–7.20 (m-3H, H aromatic at C4, C5, C6 of phenyl); 7.37–7.44 (s-1H, H aromatic at C2 of phenyl). MS-MS[ppm] = [L + H]^+^ 307.0820, found 307.0823 (0.98); [L + Na]^+^ 329.0645, [L+K]^+^ = 344.1590;

2-Chloro-1-[4-(2-methoxy)phenyl-1-piperazinyl]ethan-1-on (**3**): 

C_13_H_17_N_2_O_2_Cl (MW 268.74, MP 102–104 °C); yield 46.27%; 1H-NMR (300 MHz, CDCl3), δ (ppm) = 3.07–3.21 (t-4H, -CO-N-(CH2)2-); 3.63–3.89 (t-4H, -(CH2)2-N-C6H4-); 3.90 (s-3H, -OCH3); 4.13 (s-2H, -CH2-CO-); 6.90–6.98 (m-4H, H aromatic); MS-MS[ppm] = [L + H]^+^ 269.1051, found 269.1049 (0.74); [L + Na]^+^ 291.0871, found 291.0862 (3.09), [2L + Na]^+^ = 559.1849, found 599.1843 (1.07).

2-Chloro-1-[4-(3-methoxy)phenyl-1-piperazinyl]ethan-1-on (**4**): 

C_13_H_17_N_2_O_2_Cl (MW 268.74, MP 100–101 °C); yield 71.62%; 1H-NMR (300 MHz, CDCl3), δ (ppm) =3.03–3.30 (t-4H, -CO-N-(CH2)2-); 3.68–3.80 (t-4H, -(CH2)2-N-C6H4-); 3.91 (s-3H, -OCH3); 4.13 (s-2H, -CH2-CO-); 6.89–7.00 (m-3H, H aromatic C4, C5, C6); 7.04–7.12 (s-1H, H aromatic C2 of phenyl); MS-MS [ppm] = [L + H]^+^ 269.1051, found 269.1049 (0.74); [L + Na]^+^ 291.0871, found 291.0862 (3.09), [2L+Na]^+^ = 559.1849, found 599.1843 (1.07).

2-Chloro-1-[4-(4-methoxy)phenyl-1-piperazinyl]ethan-1-on (**5**): 

C_13_H_17_N_2_O_2_Cl (MW 268.74, MP 109–111 °C); yield 46.64%; 1H-NMR (300 MHz, CDCl3), δ (ppm) = 3.07–3.20 (t-4H, -CO-N-(CH2)2-); 3.70–3.88 (t-4H, -(CH2)2-N-C6H4-); 3.91 (s-3H, -OCH3); 4.13 (s-2H, -CH2-CO-); 6.85–7.01 (m-4H, H aromatic); MS-MS[ppm] = [L + H]^+^ 269.1051, found 269.1052 (0.37); [L + Na]^+^ 291.0871, found 291.0859 (4.12), [2L+Na]^+^ = 559.1849, found 599.1756 (16.63).

#### 3.1.3. General Method for the Preparation of the 2-[2-oxo-2-[4-arylphenyl-1-piperazinyl]]ethyl-1H-isoindole-1,3(2H)-diones **E**–**I**

A 0.007 mol amount of 1*H*-isoindole-1,3(2*H*)-dione was dissolved in 40 mL of acetonitrile, and 0.008 mol of anhydrous potassium carbonate and 0.0077 mol of 2-chloro-1-[4-(arylophenyl)-1-piperazinyl]ethanon (**1–5**) were added. The reaction mixture was refluxed for 5 h. After this time, the inorganics were filtered on corrugated filter paper. The filtrate was completely evaporated under reduced pressure. The residue was crystallized from ethanol. The properties of obtained compounds **E**–**I** are shown below in Table 5.

**E**: 2-[2-oxo-2-(4-phenyl-1-piperazinyl)ethyl]-1*H*-isoindole-1,3(2*H*)-dione:

^1^H NMR (300MHz) w CDCl3 δ [ppm] of **E**: 3.20–3.30 (t-4H, -CO-N-(C**H**2)2-); 3.71–3.90 (t-4H, -(C**H**2)2-N-C6H4-); 4.57 (s-2H, -CH2-CO-); 6.96–7.08 (m-3H, **H** aromatic at C3, C4, C5 of phenyl); 7.27–7.46 (m-2H, **H** at C2, C6 of phenyl), 7.74–7.76 (m-2H, **H** aromatic C3, C6 of phthalimide); 7.89–7.92 (m-2H, **H** aromatic at C4 i C5 of phthalimide). MS-MS[ppm] = [L + H]^+^ = 350.1499 found 305.1479 (5.71); [L + Na]^+^ = 372.1319, found 372.1300 (5.11), [2L+Na]^+^ = 721.2745, found 721.2724 (2.91).

**F**: 2-{2-oxo-2-[4-(3-trifluoromethyl)-phenyl-1-piperazinyl]ethyl}-1*H*-isoindole-1,3(2*H*)-dione:

^1^H NMR (300MHz) w CDCl3 δ [ppm] of **F:** 3.21–3.42 (t-4H, -CO-N-(CH2)2-); 3.72–3.80 (t-4H, -(CH2)2-N-C6H4-); 4.56 (s-2H, -CH2-CO-); 7.14–7.26 (m-3H, H aromatic of phenyl at C4, C5, C6); 7.38–7.44 (s-1H, H aromatic at C2); 7.73–7.56 (m-2H, H aromatic at C3,C6 of phthalimde), 7.88–7.90 (m-2H, H aromatic at C4, C5 of phthalimide). MS-MS[ppm] = [L + H]^+^ = 418.1373 found 418.1357 (3.83); [L + Na]^+^ = 440.1192, found 440.1176 (3.64); [2L + Na]^+^ = 857.2493, found 857.2485 (0.93).

**G**: 2-{2-oxo-2-[4-(2-methoxy)-phenyl-1-piperazinyl]ethyl}-1*H*-isoindole-1,3(2*H*)-dione:

^1^H NMR (300MHz) w CDCl3 δ [ppm] of **G:** 3.02–3.21 (t-4H, -CO-N-(CH2)2-); 3.68–3.87 (t-4H, -(CH2)2-N-C6H4-); 3.89 (s-3H, -OCH3); 4.51 (s-2H, -CH2-CO-); 6.89–6.95 (m-4H, H aromatic of phenyl); 7.71–7.74 (m-2H, H aromatic at C3,C6 of phthalimide); 7.87–7.90 (m-2H, H aromatic at C4, C5 of phthalimide); MS-MS[ppm] = [L + H]^+^ = 380.1605 found 380.1575 (18.41); [2L+Na]^+^ = 781.2956, found 781.2925 (3.97).

**H**: 2-{2-oxo-2-[4-(3-methoxy)-phenyl-1-piperazinyl]ethyl}-1*H*-isoindole-1,3(2*H*)-dione:

^1^H NMR (300MHz) w CDCl3 δ [ppm] of **H:** 3.08–3.60 (t-4H, -CO-N-(CH2)2-); 3.72–3.90 (t-4H, -(CH2)2-N-C6H4-); 3.92 (s-3H, -OCH3); 4.57 (s-2H, -CH2-CO-); 6.92–6.99 (m-3H, H aromatic at C4, C5, C6 of phenyl); 7.04–7.12 (s-1H, H aromatic at C2 of phenyl); 7.73–7.76 (m-2H, H aromatic at C3, C6 of phthalimide); 7.89–7.92 (m-2H, H aromatic at C4, C5 of phthalimide); MS-MS[ppm] = [L + H]^+^ = 380.1605 found 380.1573 (8.42); [L + Na]^+^ = 402.1424, found 402.1391 (5.63); [2L + Na]^+^ = 781.2956, found 781.2912 (5.63).

**I**: 2-{2-oxo-2-[4-(4-methoxy)-phenyl-1-piperazinyl]ethyl}-1*H*-isoindole-1,3(2*H*)-dione

^1^H NMR (300MHz) w CDCl3 δ [ppm] of **I:** 3.02–3.21 (t-4H, -CO-N-(CH2)2-); 3.66–3.73 (t-4H, -(CH2)2-N-C6H4-); 3.79 (s-3H, -OCH3), 4.54 (s-2H, -CH2-CO-); 6.85–6.93 (m-4H, H aromatic of phenyl); 7.71–7.74 (m-2H, H aromatic at C3, C6 of phthalimide); 7.86–7.89 (m-2H, H aromatic at C4, C5 of phthalimide); MS-MS[ppm] = [L + H]^+^ = 380.1605 found 380.1603 (0.53); [L + Na]^+^ = 402.1424, found 402.1419 (1.24); [2L + Na]^+^ = 781.2956, found 781.2959 (0.38).

### 3.2. Cell Line and Condition

The NHDF cells obtained from ATCC (Manasas, VA, USA) were incubated with DMEM without phenol red supplemented with 10% fetal bovine serum (FBS), 2 mM L-glutamine, 1.25 µg/mL amphotericin B and 100 µg/mL gentamicin at 37 °C in a humidified 5% CO_2_/95% air atmosphere incubator. The cell culture was examined under the microscope twice a week, and cells were passaged if confluence was greater than 70% or the medium was replaced with fresh medium.

### 3.3. Tested Compounds

The 10 µM stock solutions were prepared by dissolving compounds in DMSO and then stored at −20 °C for up to 6 months. Before the biological experimental, the working solution was dissolved in the medium in the concentration range of 10, 50, and 100 µM. 

### 3.4. Cell Viability

To evaluated cell viability, an MTT assay was performed. After incubation of cells with the tested compounds for 24 h, the supernatant was removed, and 1 mg/mL MTT solution was applied for 2 h. Next, the formazan was dissolved with propanol for 30 min, and absorbance was measured at 570 nm using a microplate reader. The results are presented as IC_50_. 

### 3.5. ROS and RNS Level and DNA Damage

The ROS and RNS were evaluated using DCF–DA assay and Griess reagents, respectively. First, the cells were incubated with 50 µM LPS for 24 h to induce cell stress. Then, after the cells were washed, the 100 µM tested compounds were added for 1 h. Finally, the 50 µL supernatant was transferred to a new plate and added 50 µL of mixture A (1% sulfanilamide in 5% phosphoric acid) and B (0.1% N-(1-Naphthyl)ethylenediamine dihydrochloride) Griess reagents in a volume ratio 1:1 for 20 min in the dark. After that time, the absorbance was measured at 540 nm using a microplate reader. Simultaneously, the rest of the supernatant was removed from the above cell cultures, and 25 µM DCF–DA solution in MEM (without serum and phenol red) was added and left at 37 °C for 1 h and finally, determined fluorometrically with excitation at 485 nm and emission at 535 nm using a microplate reader. 

However, the cells were detached at the surface after 1h of incubation with the tested compound for evaluating DNA damage. The cells were then subjected to an analogous procedure as described in Grajzer et al. [44].

The NO level was presented as concentration calculated using a standard curve (prepared in parallel with the conducted research). The ROS level was shown as ratio E/E0, where E0 is fluorescence with the positive control (cells incubated with 50 µM LPS) and E is a fluorescence with tested compound at 100 µM. Finally, the DNA damage was calculated as the ratio of cell nucleus diameter to halo diameter (a measure of DNA damage). 

### 3.6. Cyclooxygenase Inhibition Assay

Cayman kit No. 701050 was used to evaluate the COX peroxidase activity of all compounds at a concentration of 100 µM. Absorbance was measured with a microplate reader at 590 nm. Concentrations at which 50% inhibition of enzyme activity occurred were calculated separately for COX-1 and COX-2 (IC50). Ratios of IC50 values (COX-2/COX-1) were also calculated

### 3.7. Statistical Analysis

All statistical analysis performed were compared to the control—healthy cells (MTT assay), and positive control (incubated with 50 µM LPS) for DCF–DA, Griess, and DNA damaged. The data have a normal distribution and equality of variance, so ANOVA and post-hoc Scheffe methods were performed. The significance level was set at *p* < 0.05.

### 3.8. Molecular Docking

The ground state geometric optimizations were calculated using density functional theory (DFT) with Becke’s three-parameter hybrid exchange function with the Lee-Yang–Parr gradient corrected correlation (B3LYP) [45,46,47] functional in combination with 6–311+G (d,p) basis set. Calculations were carried out using the Gaussian 2016 A.03 software package [48]. The high-resolution crystal structure of COX-1 (2.40 Å) and COX-2 (2.45 Å) co-crystalized with Meloxicam was selected for docking studies (Protein Data Bank, PDB ID: 4O1Z, 4M11 [43]). The molecular docking study was carried out using using AutoDock 4.2.6 software (AutoDock, La Jolla, CA, USA) and AutoDock Tools 1.5.6 [49]. All the ligands and water molecules were removed, and then polar hydrogen atoms and Kollman charges were added to the protein structure. To prepare the ligand molecules, partial charges were calculated, nonpolar hydrogens were merged, and rotatable bonds were assigned. The centers of grid boxes for COX-1 and COX-2 were set according to the meloxicam binding site in the crystal structure 4O1Z and 4M11. The Lamarckian genetic algorithm was selected for the conformational search. The running times of the genetic algorithm and the evaluation times were set to 100 and 2.5 million, respectively. After the molecular docking, the ligand-receptor complexes were further analyzed using Discovery Studio Visualizer v.20, (https://www.3ds.com/, accessed on 3 June 2021)

## 4. Conclusions

New phthalimide derivatives were obtained, their structure was verified and the theoretical bioavailability was tested on the basis of descriptors according to Lipiński’s five rule. The new imides showed an affinity for both isoforms of cyclooxygenase and were non-toxic in the tested concentration range of 10–90 µM. Based on the obtained results, the COX-2/COX-1 ratio was calculated, compounds **B** and **D** (1.10 and 0.96) have a value greater than that of meloxicam (0.71), while the value for imides **G**, **C**, and **E** (0, 65; 0.67; and 0.70, respectively) is comparable to meloxicam. All compounds tested showed both ROS and NO scavenging activity. It is noteworthy that this also applies to derivatives containing a methoxy substituent on phenyl, which have cell toxicity of 0.9 to 0.92 µM. All of them also cause chromatin relaxation outside the cell nucleus. The obtained results, similar for both types of derivatives: Mannich bases (**A**–**D**) and derivatives with an ethan-1-one linker (**E**–**I**), indicate a high affinity of N-aryl piperazine-alkyl derivatives cyclooxygenase. The in vitro study results and the analysis of molecular docking of compounds to cyclooxygenase, a constitutive form of COX-1 and inflammation-induced COX-2, are consistent and comparable to the reference drug values determined in the same test. Therefore, it can be assumed that the new phthalimide derivatives are endowed with the expected analgesic and anti-inflammatory activity.

## Figures and Tables

**Table 1:** Calculated values for molar weight (MW), TPSA, logP, H-d number, and H-a number for compounds **A**–**I**.

Compound	MW (g/mol)	TPSA (Å^2^)	log*P*	H-d	H-a
**A**	321.37	45.55	3.13	0	5
**B**	351.40	54.79	3.14	0	6
**C**	389.37	45.55	4.0	0	5
**D**	411.50	45.55	4.61	0	5
**E**	349.14	60.93	2.37	0	4
**F**	417.13	60.93	3.25	0	7
**G**	379.15	70.16	2.11	0	5
**H**	379.15	70.16	2.11	0	5
**I**	379.15	70.16	2.11	0	5

**Table 2:** IC_50_ values calculated for COX-1 and COX-2 enzymes; incubation for 2 min with the tested compounds [mean (SD); *n* = 3]; statistical significance of COX-1 and COX-2 inhibition calculated with post-hoc test compared to the control Meloxicam (* *p* < 0.05). IC_50_ values were calculated for NHDF cell cultures after 24 h of incubation with the tested compounds **A**–**I**.

Compound	IC_50_ (µM) (SD)		Ratio: COX-2/COX-1	Cell Viability IC_50_ (µM) (SD)
	COX-1	COX-2		
**A**	238.3 (0.04) *	107.2 (0.09)	0.45	non-toxic
**B**	71.0 (0.03) *	68.5 (0.05)	0.96	non-toxic
**C**	104.1 (0.02) *	70.3 (0.06)	0.67	non-toxic
**D**	66.2 (0.09)	72.8 (0.07)	1.10	90.28 (0.07)
**E**	76.7 (0.09)	53.4 (0.08)	0.70	non-toxic
**F**	171.0 (0.06) *	47.6 (0.10)	0.28	non-toxic
**G**	100.7 (0.07) *	65.8(0.04) *	0.65	96.78 (0.02)
**H**	176.4 (0.07) *	43.8 (0.09)	0.25	94.12 (0.03)
**I**	94.4 (0.05) *	46.2 (0.10)	0.49	93.32 (0.05)
**Meloxicam**	83.7 (0.03)	59.2 (0.07)	0.71	-

**Table 3:** ROS and RNS scavenging activity of tested compounds and an impact on the reduction of DNA damage ((SD) *n* = 6); the results were compared to the control and expressed as E/E_0_ ratios for ROS; concentration [pg/mL] for NO and the ratio of cell nucleus diameter to halo diameter for DNA damage; statistical significance calculated with post-hoc test compared to control (* *p* < 0.05; E_0_—cultures without tested compounds).

Compound	ROS Level (E/E_0_)	NO Level (pg/mL)	DNA Damage
**Control [50 µM LPS]**	1.0 (0.03)	32.0 (0.1)	26.4 (0.08)
**A**	−18.6 (0.02) *	6.4 (0.14)	15.2 (0.04) *
**B**	−22.9 (0.04) *	4.3 (0.05) *	19.6 (0.09) *
**C**	−18.2 (0.02) *	8.2 (0.03) *	23.2 (0.07) *
**D**	−14.2 (0.08) *	7.4 (0.12)	17.3 (0.04) *
**E**	−26.2 (0.03) *	5.3 (0.07) *	18.4 (0.07) *
**F**	−22.1 (0.04) *	9.1 (0.07)	20.1 (0.03) *
**G**	−29.1 (0.09) *	4.5 (0.08)	18.7 (0.07) *
**H**	−27.6 (0.05) *	4.0 (0.05) *	17.1 (0.04) *
**I**	−28.5 (0.06) *	4.8 (0.06) *	19.2 (0.08) *

**Table 4:** Theoretical binding free energy and inhibit constant for interactions studied compounds with COX-1 and COX-2.

	ΔG° (kJmol^−1^)	K_i_ (μM)	ΔG° (kJmol^−1^)	K_i_ (μM)
	COX-1		COX-2	
**A**	−25.33	35.94	−33.10	1.56
**B**	−22.65	106.45	−31.68	2.76
**C**	−23.78	67.41	−28.05	12.13
**D**	−26.88	19.34	−30.35	4.74
**E**	−29.80	5.98	−35.20	0.68
**F**	−19.39	420.72	−30.98	3.68
**G**	−30.55	4.37	−35.74	0.54
**H**	−27.80	13.39	−32.27	2.18
**I**	−22.90	96.16	−30.47	4.50
**Meloxicam**	−33.35	1.42	−34.02	1.09

**Table 5:** The properties of obtained compounds **A**–**I**.

Comp.	Formula (MW)	M.p. (°C) Solvent	Yield (%)	TLC (Rf)	FT–IR	
				OE: MeOH 7:3	C=O	Mono-and Disubstituted Benzene
**A**	C_19_H_19_N_3_O_2_ 321.37	160–161 ethanol	76.87	0.92	1701 1770	705, 770
**B**	C_20_H_21_N_3_O_3_ 351.40	134–135 ethanol	92.91	0.78	1705 1768	727
**C**	C_20_H_18_N_3_O_2_F_3_ 389.37	147–148 ethanol	47.26	0.77	1711 1771	696, 755
**D**	C_26_H_25_N_3_O_2_ 411.50	206–207 ethanol	58.93	0.89	1705 1765	690, 750
**E**	C_20_H_19_N_3_O_3_ 349.40	180–180 ethanol	51.87	0.74	1670 1710 1750	700, 750
**F**	C_21_H_18_N_3_O_3_F_3_ 417.38	206–208 ethanol	53.59	0.79	1680 1705 1760	690, 720
**G**	C_21_H_21_N_3_O_4_ 379.41	152–154 ethanol	61.43	0.47	1640 1689 1720	700
**H**	C_21_H_21_N_3_O_4_ 379.41	155–156 ethanol	69.29	0.58	1650 1700 1730	710, 780
**I**	C_21_H_21_N_3_O_4_ 379.41	204–207 ethanol	90.71	0.53	1620 1680 1710	770

## Data Availability

The data generated and analyzed during the current study are available from the corresponding authors upon reasonable request.

## Supplementary Materials

The following are available online at https://www.mdpi.com/article/10.3390/ijms22147678/s1, The following fragmentation ions were found in the Table S1: MS/MS spectrum of compound **E: I**.; Table S2: MS/MS spectrum of compound **F: II**.; Table S3: MS/MS spectrum of compound **G: III**.; Table S4: MS/MS spectrum of compound **H: IV**.; Table S5: MS/MS spectrum of compound **I: V**.
