# Supplementary material for: A New N-Substituted 1H-Isoindole-1,3(2H)-Dione Derivative—Synthesis, Structure and Affinity for Cyclooxygenase Based on In Vitro Studies and Molecular Docking
Source: Int J Mol Sci. 2021 Jul 18;22(14):7678. doi: 10.3390/ijms22147678 (PMC8306876; doi:10.3390/ijms22147678)

Supplementary material:

I. The following fragmentation ions were found in the MS / MS spectrum of compound **E**:

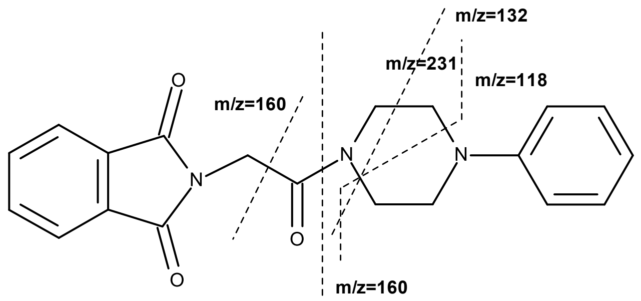

Table S1.

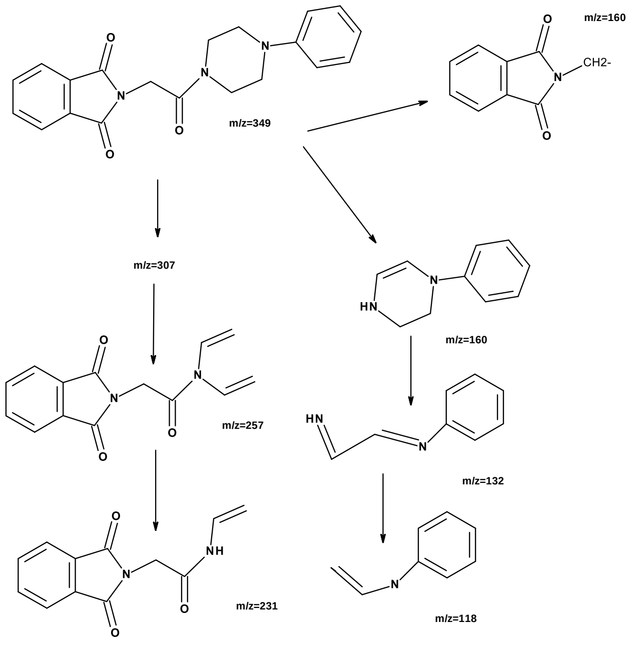

**Compound**	**MW**	**Quazi-molecular Ion adduct**	**m/z**
**Substrat E**	349	M+H	350
Fragmentation product	306	M+H	307
Fragmentation product	256	M+H	257
Fragmentation product	230	M+H	231
Fragmentation product(x2)	159	M+H	160
Fragmentation product	131	M+H	132
Fragmentation product	117	M+H	118

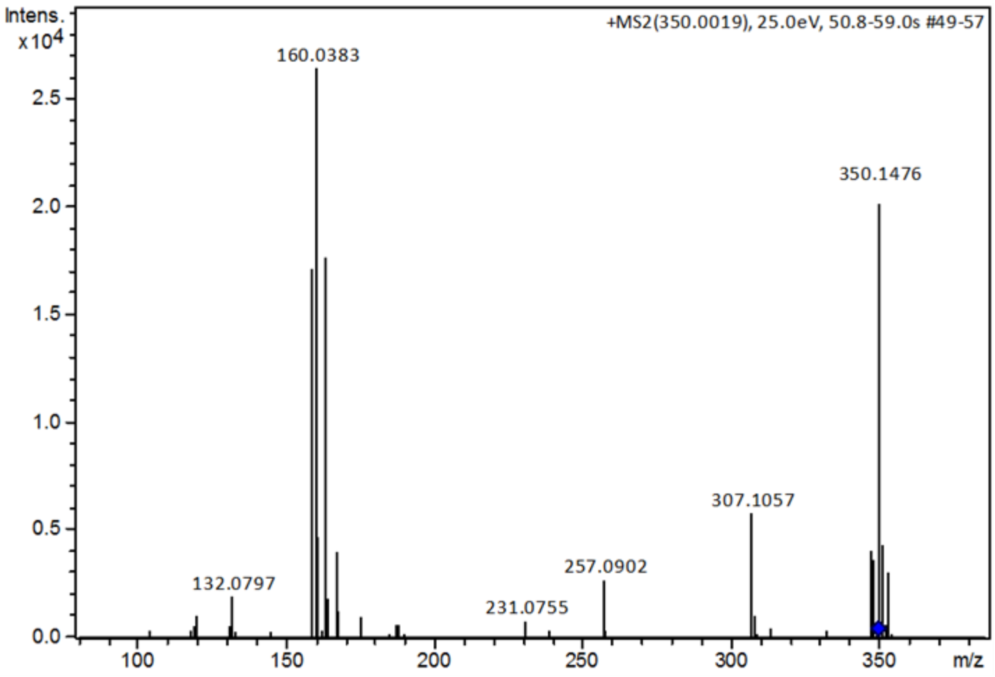
Intens.
x10^4
+MS2(350.0019), 25.0eV, 50.8-59.0s #49-57
160.0383
350.1476
307.1057
257.0902
231.0755
132.0797
2.5
2.0
1.5
1.0
0.5
0.0
100
150
200
250
300
350
m/z

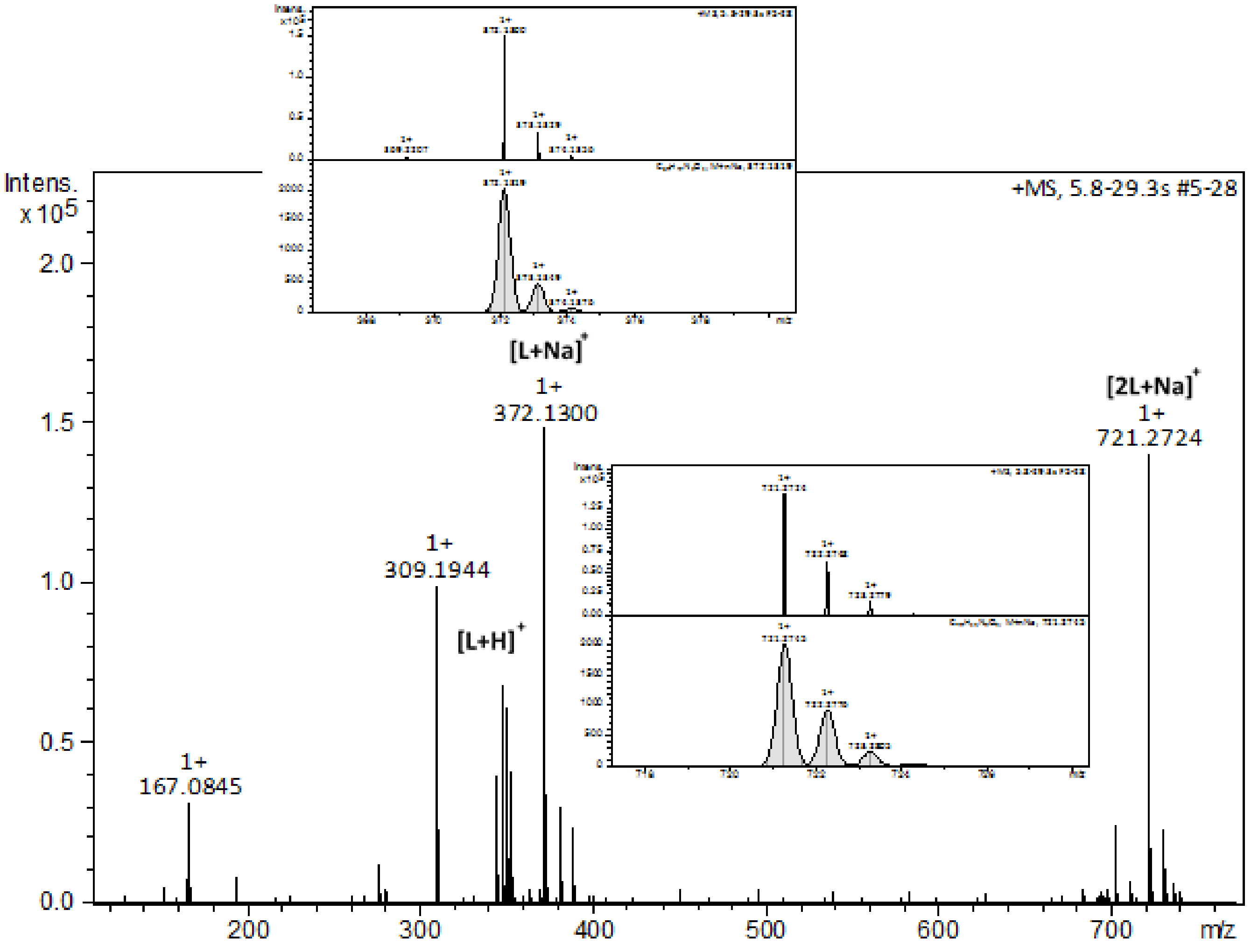
Intens.
x 10^5
+MS, 5.8-29.3s #5-28
2.0
1.5
1.0
0.5
0.0
[L+Na]+
1+
372.1300
[2L+Na]+
1+
721.2724
1+
309.1944
[L+H]+
1+
167.0845
200
300
400
500
600
700
m/z

II. The following fragmentation ions were found in the MS / MS spectrum of compound **F**:

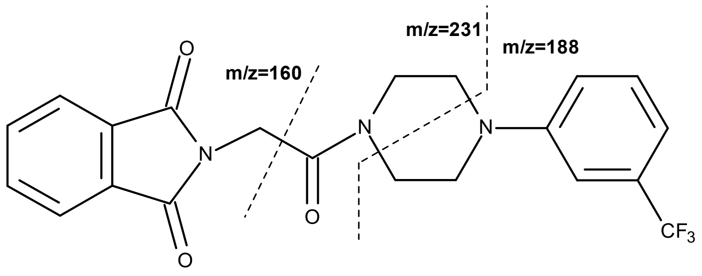

Table S2.

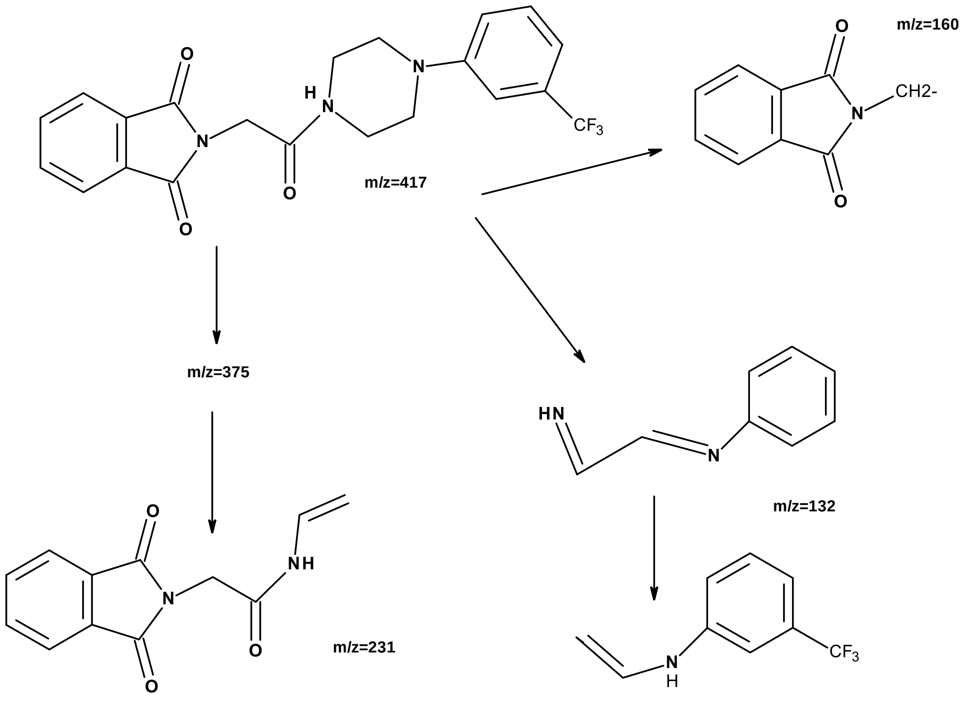

Compound	MW	Pseudo-molecular ion adduct	m/z
**Substrat F**	417	M+H	418
Fragmentation product	374	M+H	375
Fragmentation product	230	M+H	231
Fragmentation product	187	M+H	188
Fragmentation product	159	M+H	160

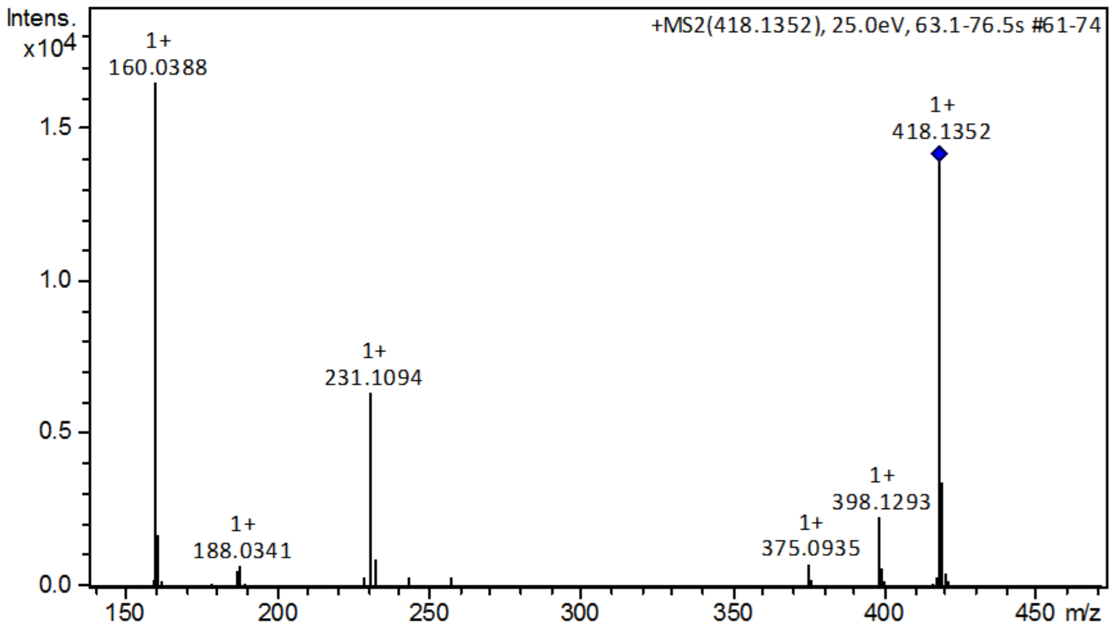
Intens.
x10^4
+MS2(418.1352), 25.0eV, 63.1-76.5s #61-74
1+
160.0388
1+
418.1352
1+
231.1094
1+
188.0341
1+
398.1293
1+
375.0935
m/z

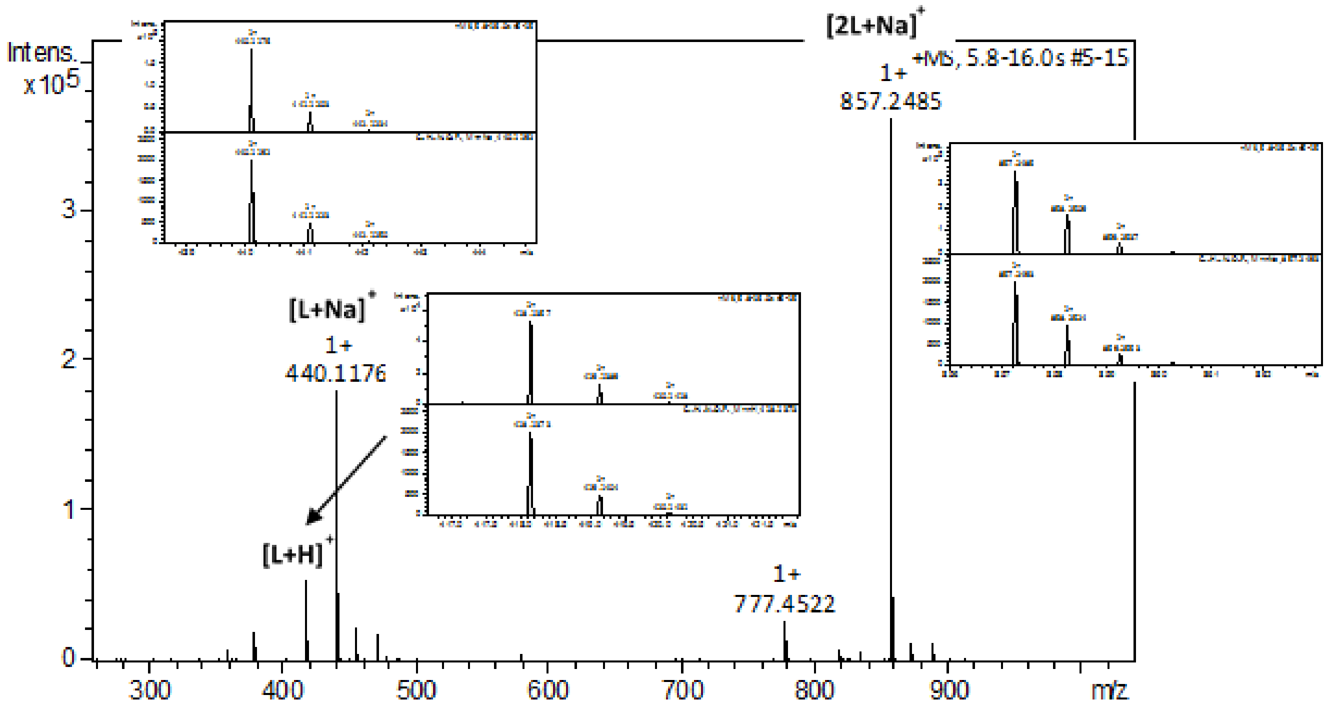
Intens.
x10^5
[2L+Na]+
+MS, 5.8-16.0s #5-15
1+
857.2485
[L+Na]+
1+
440.1176
[L+H]+
1+
777.4522
m/z

III. The following fragmentation ions were found in the MS / MS spectrum of compound **G**:

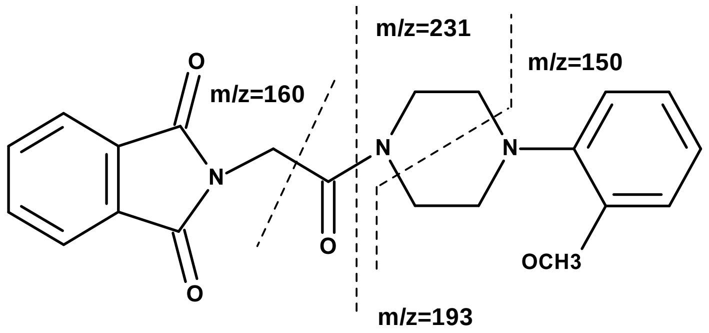

Table S3.

**Compound**	**MW**	**Quazi-molecular Ion adduct**	**m/z**
**Substrat G**	379	M+H	380
Fragmentation product	336	M+H	337
Fragmentation product	256	M+H	257
Fragmentation product	230	M+H	231
Fragmentation product	192	M+H	193
Fragmentation product	159	M+H	160
Fragmentation product	149	M+H	150

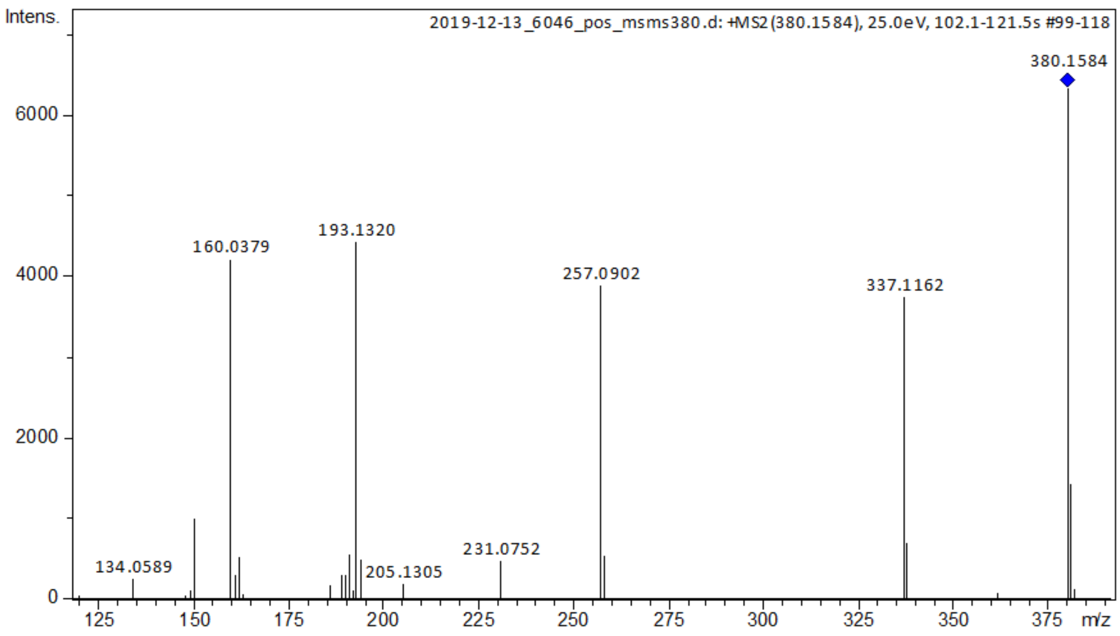

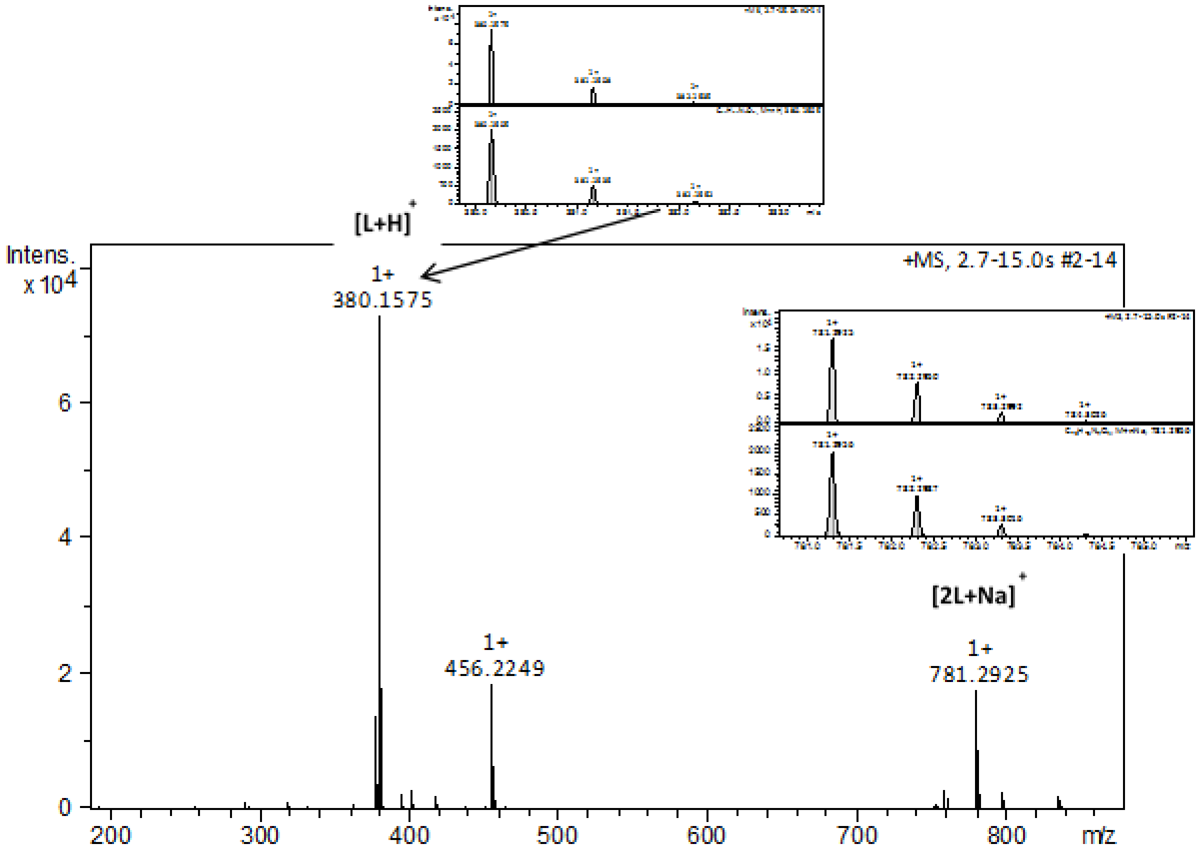
[L+H]+
Intens.
x 10^4
1+
380.1575
+MS, 2.7-15.0s #2-14
6
4
2
0
1+
456.2249
[2L+Na]+
1+
781.2925
200
300
400
500
600
700
800
m/z

IV. The following fragmentation ions were found in the MS / MS spectrum of compound **H**:

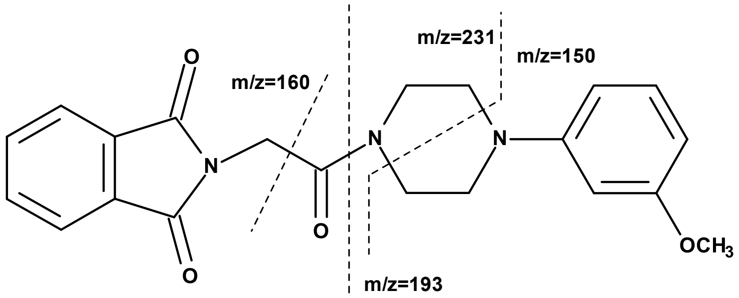

Table S4.

**Compound**	**MW**	**Quazi-molecular Ion adduct**	**m/z**
**Substrat H**	379	M+H	380
Fragmentation product	336	M+H	337
Fragmentation product	256	M+H	257
Fragmentation product	230	M+H	231
Fragmentation product	192	M+H	193
Fragmentation product	159	M+H	160
Fragmentation product	149	M+H	150

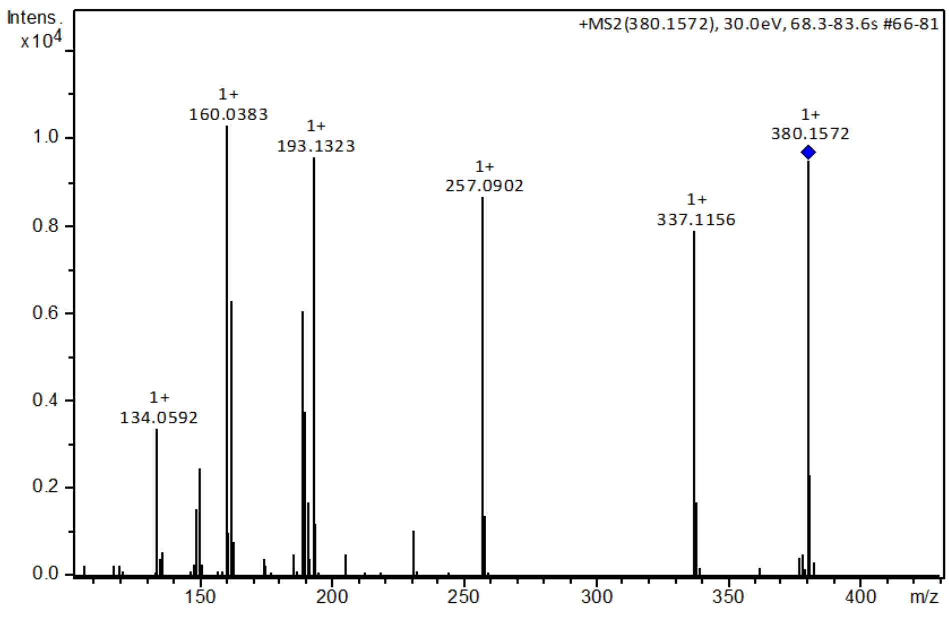

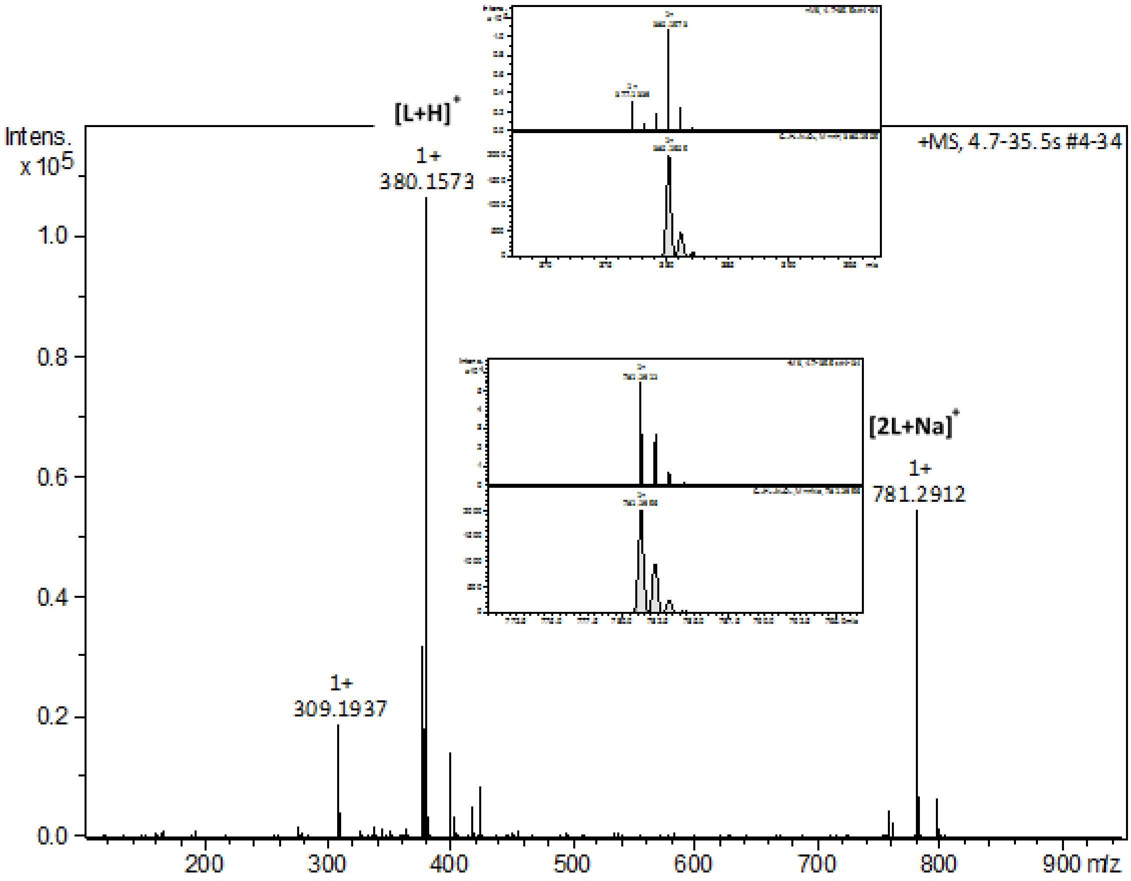

[L+H]+
1+
380.1573
[2L+Na]+
1+
781.2912
1+
309.1937
+MS, 4.7-35.5s #4-34
Intens.
x 10^5
1.0
0.8
0.6
0.4
0.2
0.0
200
300
400
500
600
700
800
900 m/z

V. The following fragmentation ions were found in the MS / MS spectrum of compound **I**:

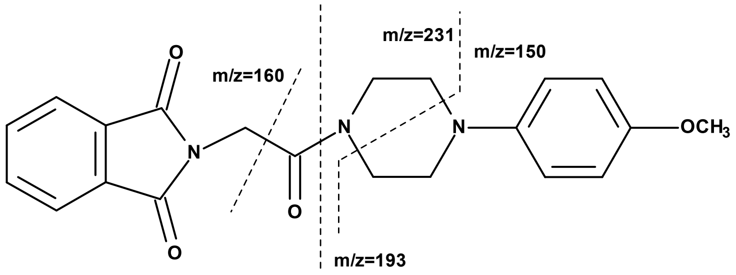

Table S5.

**compound**	**MW**	**Quazi-molecular Ion adduct**	**m/z**
**Substrat I**	379	M+H	380
Fragmentation product	336	M+H	337
Fragmentation product	256	M+H	257
Fragmentation product	230	M+H	231
Fragmentation product	192	M+H	193
Fragmentation product	159	M+H	160
Fragmentation product	149	M+H	150

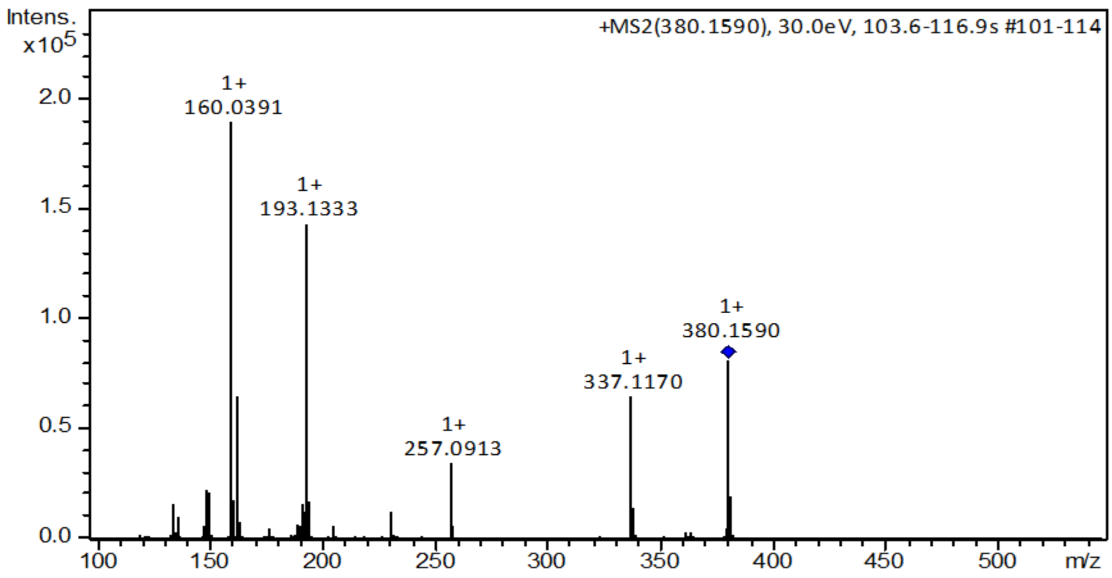

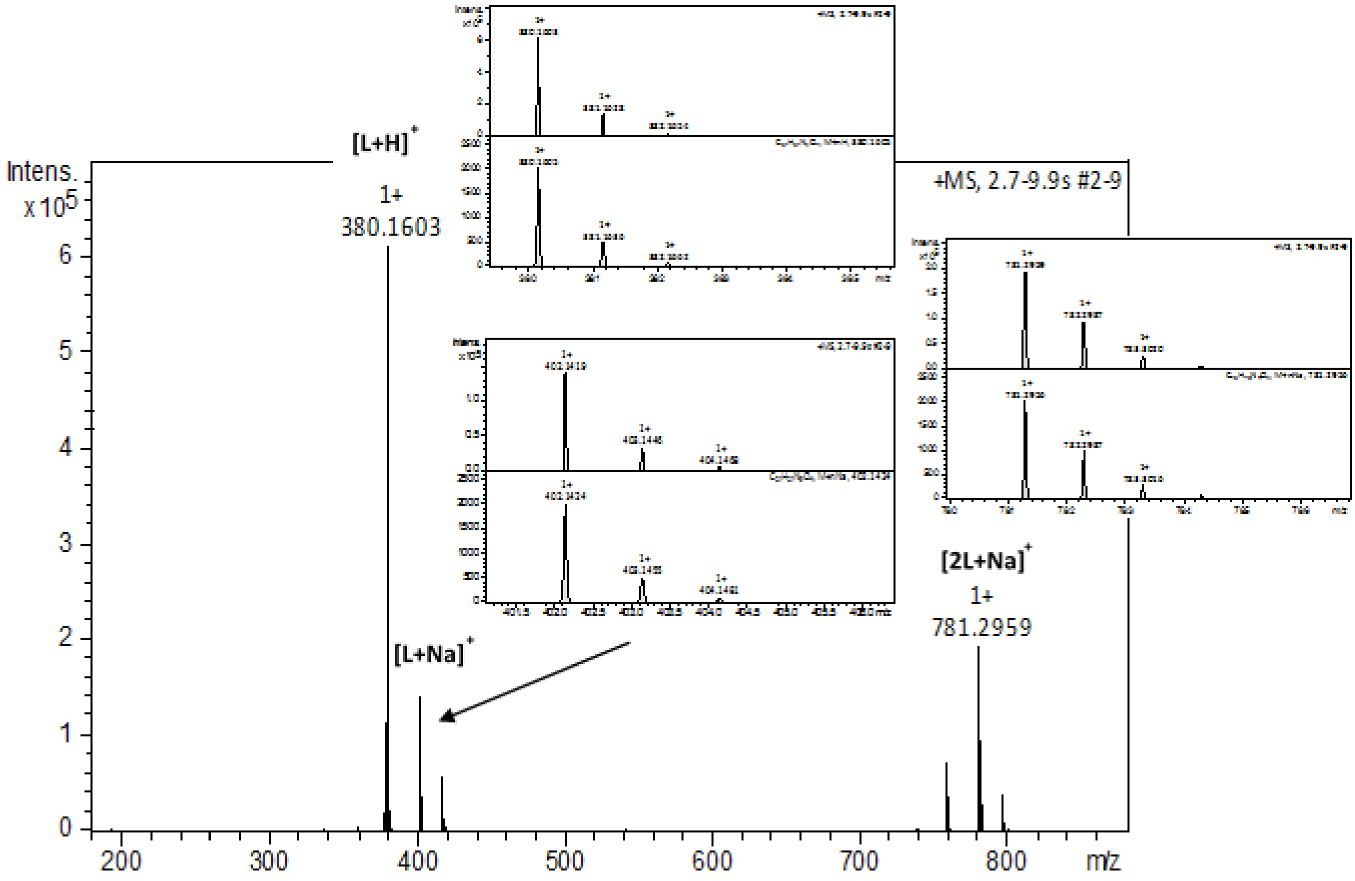

Scheme S1. The probable mechanism of G, H, I degradation in methanol solution, proposed on the basis of ESI-MS,(ad III, IV, V)

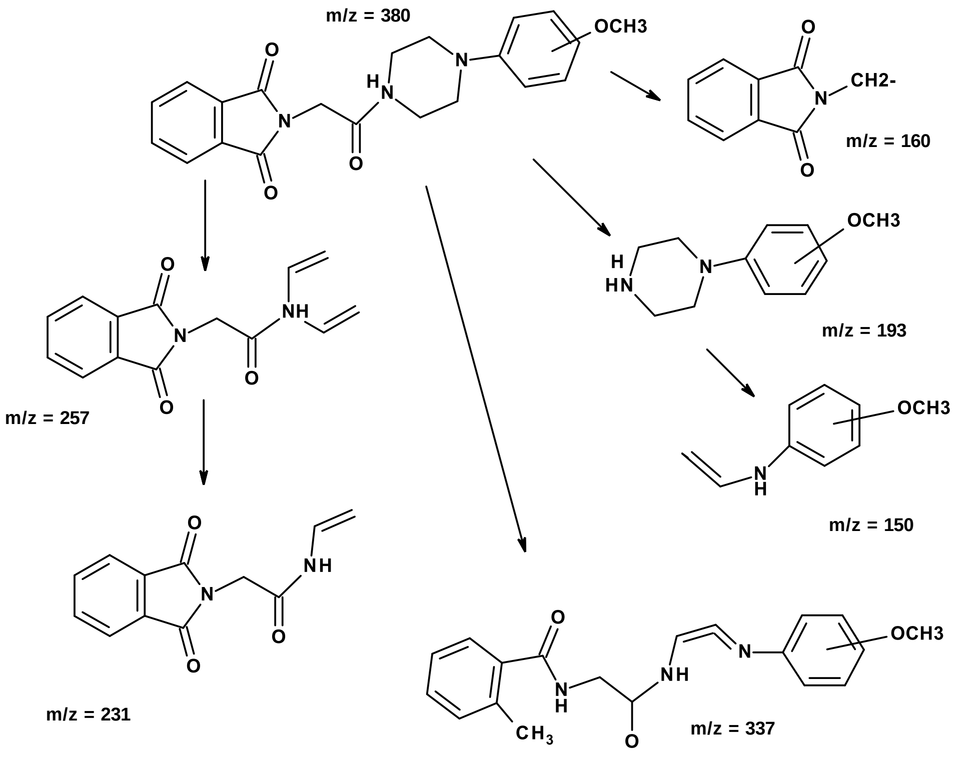

Supplement: Supplementary file 1 [file ijms-22-07678-s001.zip › ijms-1290453-supplementary.pdf]
